# Deficits in general and smoking‐specific response inhibition in the Go/No‐Go task in individuals who smoke: A cross‐sectional analysis

**DOI:** 10.1111/add.70003

**Published:** 2025-02-19

**Authors:** Franziska Motka, Simone Kühn, Charlotte E. Wittekind

**Affiliations:** ^1^ Division of Clinical Psychology and Psychotherapy, Department of Psychology LMU Munich Munich Germany; ^2^ Neuronal Plasticity Working Group, Department of Psychiatry and Psychotherapy University Medical Center Hamburg‐Eppendorf Hamburg Germany; ^3^ Center for Environmental Neuroscience Max Planck Institute for Human Development Berlin Germany

**Keywords:** addiction, covariates, cross‐sectional, Go/No‐Go task, moderators, response inhibition, smoking, tobacco dependence

## Abstract

**Background and aims:**

Previous studies on response inhibition deficits in smoking have often been conducted in small, young, age‐homogeneous samples, without controlling for covariates or testing moderating effects. The primary research question compared response inhibition between a large, age‐diverse smoking sample and non‐smoking controls, and examined whether deficits were exacerbated toward smoking‐related stimuli. By accounting for key covariates and moderators, this study aimed to extend understanding of individual differences in response inhibition deficits in smoking.

**Design and setting:**

Cross‐sectional study conducted at a university laboratory in Munich, Germany.

**Participants:**

The large (*n* = 122, 57% female), age‐diverse (*M*_age_ = 41.4, range: 21–70 years) smoking group comprised individuals with moderate to severe tobacco dependence participating in a smoking reduction intervention study. Controls comprised *n* = 69 healthy individuals with no smoking history.

**Measurements:**

Primary outcomes were commission error (CE) rates and mean reaction times in Go trials (Go‐RT) in general and smoking‐specific Go/No‐Go tasks (GNGTs). Covariates included age, sex and IQ. Smoking‐related variables were cigarettes per day (CPD), tobacco dependence severity and craving.

**Findings:**

General GNGT: The smoking group exhibited significantly higher CE rates (*P*‐value < 0.001, medium effect, BF_10_ = 9.06) than the control group. Higher craving was associated with faster Go‐RTs (*β* = −1.487, *P*‐value = 0.041). Smoking‐specific GNGT: CE rates were significantly higher in the smoking group only when controlling for covariates (*β* = 1.272, *P*‐value = 0.040). Higher craving was associated with higher CE rates during smoking‐related trials (*β* = 0.108, *P*‐value = 0.010). The smoking group showed significantly faster Go‐RTs in response to smoking‐related compared with neutral stimuli, relative to the control group (*β* = −3.326, *P*‐value = 0.027). Preliminary evidence indicated that greater deficits were associated with higher scores in smoking‐related variables, but only in older individuals.

**Conclusions:**

Individuals who smoke appear to exhibit response inhibition deficits, although these are not uniform and seem to be exacerbated during higher reported craving or in response to smoking‐related stimuli. Age may moderate the relationship between deficits and smoking‐related variables.

## INTRODUCTION

Dual‐process models of addiction [1, 2] underscore the significance of impaired executive functioning in the development and maintenance of problematic substance use. The incentive‐sensitization theory (IST) [3] further suggests that these deficits are exacerbated following drug‐induced neurobiological changes in the mesocorticolimbic reward system. Continued drug use results in sensitization to the incentive salience (i.e. ‘wanting’) of drug‐related cues, which manifests in heightened responsivity to these cues.

Response inhibition, a core executive function, is the ability to suppress pre‐potent behaviour in the presence of ‘stop’ cues [4]. It has frequently been assessed using the Go/No‐Go task (GNGT) [5], where participants respond quickly to frequent ‘Go’ stimuli and inhibit responses to infrequent ‘No‐Go’ stimuli. Deficits in response inhibition are primarily evaluated by the rate of commission errors (CE), which refers to failures to inhibit responses during No‐Go trials. Faster mean reaction times in Go trials (Go‐RTs) indicate higher responsivity [4, 6].

Meta‐analytical results confirmed that individuals who smoke show higher CE rates in the GNGT [4]. So far, six cross‐sectional studies (including [7, 8, 9, 10, 11, 12]) used the GNGT including smoking‐related stimuli to test the assumptions of the IST (i.e. higher CE rates and faster Go‐RTs toward smoking‐related stimuli). The findings are mixed. Some studies confirmed that individuals who smoke exhibit higher CE rates [9, 12] 1 alongside shorter Go‐RTs [12] in smoking‐related compared to neutral trials. However, other studies did not observe higher CE rates [8, 10] 2 or only reported shorter Go‐RTs [8]. Inconsistencies may result from variations in the implementation of smoking‐related contexts: whereas Kräplin *et al*. [8] and Luijten *et al*. [10] used randomly interleaved smoking‐related and neutral trials, Li *et al*. [9] and Tsegaye *et al*. [12] used a block design.

Although several studies used GNGTs in smoking samples, previous research has significant limitations. First, most studies had small sample sizes, both in general GNGT studies [4] (*n* < 30 individuals who smoke [13, 14], but see [15]) and smoking‐specific GNGT studies (*n* < 30 individuals who smoke [7, 8, 10, 11]), which increases the likelihood of Type‐I and Type‐II errors [16]. Second, most studies focused on young samples (general GNGT: see tables 1 and 2 in [4], *M*_age_ < 30 [13, 14], but see [15]; smoking‐specific GNGT: *M*_age_ < 30 [7, 8, 9, 10, 11, 12]), limiting the generalizability of findings to older individuals who smoke. Third, most GNGT studies did not control for important covariates, such as age, sex, or intelligence [7, 8, 9, 10, 11, 17]. However, these variables are known to be associated with both smoking‐related variables (i.e. tobacco dependence severity, craving [18, 19] 3) and GNGT performance [20, 21, 22], and should be included as potential confounders in analyses [23]. Fourth, to our knowledge, no study has yet examined moderators on the relationship between smoking‐related variables and GNGT performance. However, this is important, because individuals can differ in their ability to compensate for deficits and their strategy to balance speed and accuracy (speed‐accuracy trade‐off [4, 6]). Older adults and females may prioritize accuracy [24, 25], whereas individuals with higher intelligence might better compensate for inhibitory deficits [26].

Given these limitations, the objective of the present study was to extend prior research on response inhibition deficits among individuals who smoke. Both a general and smoking‐specific GNGT—the latter adapted from Luijten *et al*. [10] with interleaved design—were used. The research questions were: (1) to examine group differences in GNGT performance (i.e. CE rates and Go‐RTs) using *t* tests and analyses of variance (ANOVAs) without accounting for covariates to facilitate comparisons with prior GNGT studies, hypothesizing that the smoking group would show higher CE rates than the non‐smoking group; (2) to assess the robustness of group differences by including age, sex and intelligence as covariates in the regression models; and (3) to investigate associations between smoking‐related variables [i.e. cigarettes per day (CPD), tobacco dependence severity and craving] and GNGT performance in individuals who smoke, hypothesising that higher scores in smoking‐related variables would be associated with higher CE rates. Additionally, we exploratory investigated whether age, sex and intelligence moderate the association between smoking‐related variables and GNGT performance. Following the assumptions of the IST, we expected individuals who smoke to exhibit higher CE rates and faster Go‐RTs toward smoking‐related stimuli compared to non‐smoking individuals, and within the smoking group, among those with higher scores in smoking‐related variables.

## METHODS

The data and analysis code is available in Open Science Framework: https://osf.io/rxu78/ (dataset) [27]. The analyses were pre‐registered (see AsPredicted.org, 172127, 24 April 2024), except for the exploratory moderator analyses. For additional methodological information see Appendix A.

### Participants and design

A total of 122 non‐deprived smoking adults with no substance use disorder other than moderate to severe tobacco dependence [i.e. ≥ 3 in the Fagerström Test for Nicotine Dependence (FTND) [28]], who participated in an intervention study [29] aiming at reducing smoking behaviour completed the baseline assessment and were included in the present cross‐sectional study (for more information, see Appendix A.1). After conducting an *a priori* power analysis (see Appendix A.2), a total of 69 age‐matched healthy individuals with no history of smoking (i.e. ≤ 10 cigarettes smoked in lifetime) or any other substance use disorder were recruited as controls. For full eligibility criteria and a critical discussion of our inclusion/exclusion criteria, see Appendices A.1 and A.1.1. The study was approved by the Ludwig‐Maximilians‐University Munich ethics committee (72_Wittekind_c). All participants provided written informed consent.

### Procedure and measures

A series of interviews, questionnaires and experimental tasks were conducted (see Appendix A.3). Basic drug‐related information was collected during the initial interview (e.g. CPD or lifetime smoking). Tobacco dependency was evaluated using the 6‐item FTND [28]. Craving was assessed through the Questionnaire of Smoking Urges, brief version (QSU‐brief [30]), a 10‐item questionnaire on current smoking urges using a 7‐item Likert‐like scale. General intelligence was screened with a German vocabulary test [Wortschatztest (WST) [31]], with raw scores converted to intelligence quotient (IQ) scores per the manual. To assess alcohol drinking behaviour, the 10‐item Alcohol Use Disorder Identification Test (AUDIT [32]) was used.

Two GNGTs were used: a general (stimuli: digits) and a smoking‐specific (stimuli: smoking‐related and neutral pictures) version. Both tasks were implemented with 320 test trials, a Go:No‐Go trial ratio of 75:25 and a response window/stimulus presentation time of 1000 ms (see Appendix A.3.1 for a detailed task description).

### Statistical analysis

#### Data pre‐processing, aggregation and reliability

As pre‐registered, CE rates (in %) and mean Go‐RTs were calculated as primary outcomes. Because no participant reached an omission error (OE) rate above 35%, all GNGT data were included in the analysis. Split‐half reliabilities for CE rates and Go‐RTs were estimated at r ≥ 0.609 (see Appendix A.4.1 for details). Missing data were limited to QSU‐brief scores relevant to research question 3 [3 missing values of 122 (~2.46%)]. No imputation was performed for the main analysis. The results remained consistent in a sensitivity analysis conducted after data imputation (see Appendix A.4.1).

#### Strategy of data analysis

Data were analysed using R, version 4.3.0 (R Core Team, 2023). As GNGT data violated conventional test and model assumptions (e.g. influential data points such as outliers and high leverage points, see Appendix A.4.2), robust statistical methods were used following recommendations by Field and Wilcox [33]. These methods are less sensitive to influential data points and other data issues that could bias model parameter estimates. For more details on the analysis strategy (e.g. R packages, data trimming procedure) see Appendix A.4.2.

For research question 1, robust two‐sample *t* tests (general GNGT) and mixed‐effects ANOVAs (smoking‐specific GNGT) on trimmed means were performed to examine group differences in GNGT performance measures. Additionally, Bayes factors (BF_10_) were calculated to complement the inference statistics by quantifying the relative evidence for both the null (e.g. no group effect) and the alternative (e.g. presence of a group effect) hypotheses [34]. For research question 2, robust multiple linear regressions were conducted to examine whether group (smoking/non‐smoking) was associated with general GNGT performance while controlling for age, sex and IQ (covariates). The smoking‐specific GNGT data was analysed using robust linear mixed‐effects models, with stimulus type (smoking‐related/neutral) and stimulus type × group as additional predictors. For research question 3, the performance of the smoking group in the general GNGT was examined by conducting three robust multiple linear regressions with either CPD, the FTND or QSU‐brief score (smoking‐related variables) as main predictor. Each model included age, sex and IQ as covariates and their interactions with the respective main predictor (exploratory moderator analyses). For the smoking‐specific GNGT, again, robust linear mixed‐effects models were used, including stimulus type and its interaction with CPD, FTND or QSU‐brief as additional predictors (exploratory moderator analyses). Additionally, analyses for research question 3 were conducted with smoking duration, rather than age, as predictor variable (see Appendix D).

Following dual‐process models [1, 2], smoking status and higher scores in smoking‐related variables should be associated with greater deficits in response inhibition (i.e. higher CE rates). In line with the IST [3], smoking status and higher scores in smoking‐related variables are expected to be associated with exacerbated responsivity (i.e. higher CE rates and faster Go‐RTs) to smoking‐related stimuli. Therefore, directional hypotheses with one‐sided *P*‐values for statistical inference were tested regarding the following effects: effects of group and smoking‐related variables on CE rates, and effects of group × stimulus type and smoking‐related variables × stimulus type on CE rates and Go‐RTs. The Benjamini‐Hochberg correction [35] was applied to control the false discovery rate (FDR) at 5% (see Appendix A.4.2).

## RESULTS

For results on OE rates see Table 2 and Appendix C.

### Description of study groups

The characteristics of both study groups are presented in Table 1. Participants who smoked consumed an average of 19.55 CPD (SD = 9.34; range: 7–60), for 22.28 years (SD = 13.20; range: 1–56), and exhibited moderate tobacco dependence [FTND: mean (M) = 5.23, SD = 1.85, range: 3–10]. The smoking group demonstrated significantly lower IQ scores, a lower proportion of participants with a high school degree and a higher, 4 albeit still low‐risk (AUDIT score < 8 [32]) alcohol consumption compared to the control group.

**TABLE 1:** Demographic and smoking‐related variables by study group.

Variables	Smoking group (*n* = 122)			Control group (*n* = 69)			*P*‐value
	M	SD	Range	M	SD	Range	
Demographic							
Age (y)	41.4	13.0	21–70	41.8	12.5	20–67	0.834
Female^a^ (*n*, %)	70, 57%			46, 67%			0.268
High school degree (*n*, %)	78, 64%			62, 90%			**<0.001**
WST‐IQ	104.85	11.01	78–139	108.96	9.12	86–125	**0.009**
AUDIT^b^	6.15^e^	4.58	0–21	2.45	1.95	0–7	**<0.001**
Smoking‐related							
Cigarettes lifetime	–	–	–	3.07	3.30	0–10	–
Cigarettes per day (*n*)	19.55	9.34	7–60	–	–	–	–
Smoking duration (years)	22.28	13.20	1–56	–	–	–	–
CO value	22.64	10.65	10–54	–	–	–	–
FTND^c^	5.23	1.85	3–10	–	–	–	–
QSU‐brief^d^	18.68^f^	10.03	0–47	–	–	–	–

*Note*: Baseline differences of continuous and categorical variables were assessed using chi‐square and two‐sample *t* tests. Significant *P*‐values are printed in bold.

Abbreviations: AUDIT, Alcohol Use Disorders Identification Test; CO, carbon monoxide; FTND, Fagerström Test for Nicotine Dependence; QSU‐brief, Questionnaire on Smoking Urges, brief version; WST, Wortschatztest (German vocabulary test); IQ, intelligence quotient.

^a^ Collected on the concept of sex. Total score ranges:

^b^ 0–40,

^c^ 0–10,

^d^ 0–60.

^e^ *n* = 104 and

^f^ *n* = 119, because of missing values during data acquisition (see Appendix A.4.1).

### Research question 1: Group difference analyses: Smoking versus control group

#### Effects of group and stimulus type on CE rates

In the general GNGT, the smoking group exhibited a significantly higher CE rate than the control group, *T*_*y*_ *=* −3.13, *P*‐value < 0.001, *P*‐value_FDR_ = 0.002, 95% CI = −6.39 to 1.66, ξ = 0.33 5 (see Table 2), supported by Bayesian analyses (BF_10_ = 9.06 6) indicating substantial evidence for higher CE rates in the smoking group. In the smoking‐specific GNGT, the ANOVA did not show evidence for a group effect on CE rates, *Q*(1,78.51) = 3.38, *P*‐value = 0.070, *P*‐value_FDR_ = 0.104, ξ = 0.24. In contrast, Bayesian analyses (BF_10_ = 1.88) indicated weak evidence for higher CE rates in the smoking group, meaning no clear conclusion can be drawn. The main effect of stimulus type lacked statistical significance on CE rates, *Q*(1,83.45) = 3.49, *P*‐value = 0.065, ξ = 0.10. There was no support for a significant interaction between group and stimulus type on CE rates, *Q*(1,83.45) = 0.87, *P*‐value = 0.354, *P*‐value_FDR_ = 0.354.

**TABLE 2:** Means, SDs and medians of CE rates, OE rates and Go‐RTs in both GNGTs by study group.

Variables	Smoking group (*n* = 122)			Control group (*n* = 69)		
	M	SD	Median	M	SD	Median
General GNGT						
CE rate	17.25	10.76	16.25	13.19	8.55	11.25
OE rate	1.08	3.09	0.42	0.60	1.14	0.00
Go‐RT	472.67	51.93	463.06	464.66	40.55	456.42
Smoking‐specific GNGT						
All trials						
CE rate	6.09	6.36	3.75	4.35	4.83	3.75
OE rate	0.20	0.63	0.00	0.17	0.32	0.00
Go‐RT	416.17	59.65	396.52	393.39	38.49	394.50
Smoking‐related trials						
CE rate	6.21	6.15	5.00	4.46	5.20	2.50
OE rate	0.19	0.60	0.00	0.16	0.46	0.00
Go‐RT	415.53	59.75	399.11	394.74	39.23	395.32
Neutral trials						
CE rate	5.96	7.76	3.75	4.24	5.29	2.50
OE rate	0.22	0.74	0.00	0.18	0.37	0.00
Go‐RT	416.80	60.07	395.33	392.04	38.40	391.30

Abbreviations: CE, commission error; GNGT, Go/No‐Go task; Go‐RT, mean reaction time in Go trials; OE, omission error.

#### Effects of group and stimulus type on Go‐RTs

In the general GNGT, the result of the *t* test did not provide evidence for a difference in Go‐RTs between groups, *T*_*y*_ = −1.05, *P*‐value = 0.282, *P*‐value_FDR_ = 0.282, 95% CI = −23.52 to 7.27, ξ = 0.12, supported by Bayesian analyses (BF_10_ = 0.29) indicating substantial evidence in favour of no group difference. Accordingly, Go‐RTs were similar in both the smoking and non‐smoking groups. In the smoking‐specific GNGT, a significant main effect of group (non‐significant after FDR‐correction) was observed on Go‐RTs, *Q*(1,82.33) = 4.32, *P*‐value = 0.041, *P*‐value_FDR_ = 0.123, ξ = 0.26, indicating that the smoking group tended to show slower RTs across all Go trials. Bayesian analyses (BF_10_ = 6.79) provided substantial evidence for a group difference, supporting the finding of slower Go‐RTs in the smoking group. The main effect of stimulus type lacked statistical significance on Go‐RTs, *Q*(1,83.43) = 0.61, *P*‐value = 0.437, ξ = 0.02. There was no statistical support for a significant interaction between group and stimulus type on Go‐RT, *Q*(1,83.43) = 2.57, *P*‐value = 0.113, *P*‐value_FDR_ = 0.169.

### Research question 2: Regression analyses with group and covariates as predictors

The results of research question 2 serve as a robustness check for those of research question 1, as the regression models controlled for important covariates including age, sex and IQ.

#### Effects of group and stimulus type on CE rates

The smoking group showed significantly higher CE rates in both GNGTs compared to the control group (see Table 3; smoking‐specific GNGT: non‐significant after FDR‐correction). Notably, when the regression model for the smoking‐specific GNGT was conducted without covariates, the group difference in CE rates was no longer significant. In the smoking‐specific GNGT, no significant interaction between group and stimulus type on CE rates was observed.

**TABLE 3:** Results of regression models on CE rates and Go‐RTs in both GNGTs.

Predictors	CE rate			Go‐RT		
	β	95% CI	*P*‐value	β	95% CI	*P*‐value
General GNGT						
Intercept	13.436	10.763–16.109	**<0.001**	469.040	455.607–482.474	**<0.001**
Group (1: smoking)	3.217	0.620–5.814	**0.008** ^a^	4.548	−8.504 to 17.599	0.493
Age	−0.189	−0.289 to −0.088	**<0.001**	1.473	0.969–1.978	**<0.001**
Sex (1: female)	−1.301	−3.828 to 1.226	0.311	−7.923	−20.623 to 4.776	0.220
IQ	−0.072	−0.197 to 0.052	0.253	−0.219	−0.845 to 0.407	0.491
Smoking‐specific GNGT						
Intercept	3.254	1.857–4.651	**<0.001**	391.031	375.883–406.178	**<0.001**
Group (1: smoking)	1.272	−0.154 to 2.698	**0.040** ^b^	17.533	2.787–32.279	**0.020** ^a^
Age	−0.102	−0.151 to −0.054	**<0.001**	1.680	1.113–2.247	**<0.001**
Sex (1: female)	0.502	−0.709 to 1.713	0.417	2.977	−11.299 to 17.254	0.683
IQ	0.011	−0.048 to 0.071	0.712	−0.840	−1.544 to −0.136	**0.019**
Stimulus type (1: smoking)	0.333	−0.779 to 1.446	0.557	2.443	0.093–4.792	**0.042**
Stimulus type × group	0.175	−1.217 to 1.567	0.403	−3.326	−6.265 to −0.386	**0.027** ^a^

*Note*: For the effects of group and stimulus type × group on CE rates, one‐sided *P*‐values are reported, otherwise two‐sided. All regression model predictors, except for sex, were grand‐mean centred. Significant *P*‐values are printed in bold.

Abbreviations: CE, commission error; GNGT, Go/No‐Go task; Go‐RT, mean reaction time in Go trials; IQ, intelligence quotient.

^a^ Significant after Benjamini‐Hochberg correction.

^b^ No effect of group was observed in a regression model without covariates (i.e. age, sex and IQ).

#### Effects of group and stimulus type on Go‐RTs

In the general GNGT, the effect of group on Go‐RTs was inconclusive. In the smoking‐specific GNGT, the smoking group exhibited significantly slower Go‐RTs compared to the control group. There was a significant interaction between group and stimulus type on Go‐RTs, indicating that the smoking group exhibited faster mean RTs in smoking‐related compared to neutral Go trials, whereas the control group showed faster mean RTs in neutral Go trials (see Table 2).

#### Effects of covariates on CE rates and Go‐RTs

Across both GNGTs, CE rates decreased with age, whereas Go‐RTs increased significantly. There was no clear evidence indicating a relationship between sex and CE rates or Go‐RTs. In the smoking‐specific GNGT, a higher IQ was associated with faster Go‐RTs, whereas no association was observed with Go‐RTs in the general GNGT.

### Research question 3: Regression analyses with smoking‐related variables and covariates in individuals who smoke

#### Effects of smoking‐related variables and stimulus type on CE rates and Go‐RTs

Smoking‐related variables (i.e. CPD, FTND and QSU‐brief) did not show conclusive evidence of a relationship with performance in the GNGTs (see Tables 4 and 5), with one exception: higher QSU‐brief scores were associated with faster Go‐RTs in the general GNGT (non‐significant after FDR‐correction).

**TABLE 4:** Results of regression models on CE rates and Go‐RTs in the general GNGT.

Predictors	CPD			FTND			QSU‐brief^a^		
	β	95% CI	*P*‐value	β	95% CI	*P*‐value	β	95% CI	*P*‐value
CE rate									
Intercept	15.785	13.252–18.318	**<0.001**	16.169	13.446–18.892	**<0.001**	16.232	13.637–18.826	**<0.001**
Age	−0.209	−0.346 to −0.072	**0.003**	−0.220	−0.366 to −0.074	**0.003**	−0.185	−0.329 to −0.041	**0.012**
Sex (1: female)	−0.210	−3.505 to 3.086	0.900	−0.509	−4.075 to 3.058	0.778	0.040	−3.427 to 3.508	0.982
IQ	−0.102	−0.254 to 0.049	0.184	−0.091	−0.257 to 0.075	0.281	−0.026	−0.189 to 0.137	0.753
Variable	−0.133	−0.409 to 0.142	0.830	−0.246	−1.758 to 1.267	0.626	−0.021	−0.302 to 0.260	0.559
Variable × age	0.024	0.009–0.039	**0.002** ^b^	0.072	−0.002 to 0.146	0.060	0.016	0.001–0.030	**0.040**
Variable × sex	0.315	−0.037–0.667	0.079	0.566	−1.376 to 2.509	0.565	0.051	−0.297 to 0.399	0.772
Variable × IQ	−0.002	−0.020 to 0.016	0.831	−0.012	−0.106 to 0.081	0.799	−0.010	−0.027 to 0.007	0.261
Go‐RT									
Intercept	478.303	465.424–491.183	**<0.001**	477.138	463.679–490.598	**<0.001**	476.473	463.316–489.630	**<0.001**
Age	1.668	0.971–2.365	**<0.001**	1.554	0.834–2.274	**<0.001**	1.742	1.012–2.472	**<0.001**
Sex (1: female)	−10.374	−27.130 to 6.383	0.223	−9.418	−27.049 to 8.213	0.292	−9.156	−26.743–8.430	0.304
IQ	−0.362	−1.132 to 0.409	0.355	−0.421	−1.243 to 0.401	0.312	−0.553	−1.382 to 0.276	0.189
Variable	0.028	−1.372 to 1.429	0.968	4.436	−3.040 to 11.911	0.242	−1.487	−2.912 to −0.062	**0.041**
Variable × age	−0.072	−0.148 to 0.004	0.064	−0.263	−0.630 to 0.104	0.158	−0.024	−0.099 to 0.052	0.534
Variable × sex	−0.228	−2.017 to 1.562	0.801	−2.760	−12.362 to 6.842	0.570	1.662	−0.101 to 3.425	0.064
Variable × IQ	0.075	−0.017 to 0.167	0.110	−0.092	−0.555 to 0.370	0.693	0.061	−0.025 to 0.147	0.164

*Note*: The predictor variable refers to either CPD, FTND or QSU‐brief (smoking‐related variables). For the effects of smoking‐related variables on CE rates, one‐sided *P*‐values are reported, otherwise two‐sided. All regression model predictors, except for sex, were grand‐mean centred. Significant *P*‐values are printed in bold.

Abbreviations: CE, commission error; CPD, cigarettes per day; FTND, Fagerström Test for Nicotine Dependence; Go‐RT, mean reaction time in Go trials; QSU‐brief, Questionnaire on Smoking Urges, brief version; IQ, intelligence quotient.

^a^ *n* = 119, because of missing values during data acquisition (see Appendix A.4.1). After data imputation, the results remained unchanged.

^b^ Significant after Benjamini‐Hochberg correction.

**TABLE 5:** Results of regression models on CE rates and Go‐RTs in the smoking‐specific GNGT.

Predictors	CPD			FTND			QSU‐brief^a^		
	β	95% CI	*P*‐value	β	95% CI	*P*‐value	β	95% CI	*P*‐value
CE rate									
Intercept	4.535	3.195–5.874	**<0.001**	4.506	3.165–5.846	**<0.001**	4.756	3.398–6.115	**<0.001**
Age	−0.120	−0.188 to −0.052	**0.001**	−0.133	−0.201 to −0.066	**<0.001**	−0.137	−0.208 to −0.066	**<0.001**
Sex (1: female)	0.360	−1.274 to 1.995	0.666	0.376	−1.273 to 2.026	0.655	−0.077	−1.790 to 1.636	0.930
IQ	−0.024	−0.100 to 0.051	0.523	−0.014	−0.090 to 0.063	0.729	−0.024	−0.104 to 0.057	0.564
Variable	−0.062	−0.208 to 0.083	0.799	−0.110	−0.852 to 0.633	0.614	−0.014	−0.160 to 0.132	0.575
Variable × age	0.009	0.002–0.017	**0.015**	0.046	0.012–0.081	**0.008**	−0.003	−0.010 to 0.004	0.416
Variable × sex	0.031	−0.144 to 0.205	0.730	−0.492	−1.390 to 0.407	0.283	−0.059	−0.230 to 0.113	0.503
Variable × IQ	−0.008	−0.017 to 0.001	0.067	−0.033	−0.076 to 0.010	0.133	−0.006	−0.015 to 0.002	0.150
Stimulus type (1: smoking)	0.516	−0.415 to 1.447	0.277	0.490	−0.428 to 1.409	0.296	0.440	−0.463 to 1.342	0.340
Variable × stimulus type	0.003	−0.097 to 0.103	0.476	0.036	−0.463 to 0.535	0.444	0.108	0.017–0.198	**0.010**
Go‐RT									
Intercept	413.749	398.828–428.670	**<0.001**	410.501	395.795–425.207	**<0.001**	409.127	394.056–424.198	**<0.001**
Age	2.121	1.315–2.927	**<0.001**	2.141	1.355–2.926	**<0.001**	2.347	1.513–3.182	**<0.001**
Sex (1: female)	7.991	−11.383 to 27.366	0.419	9.575	−9.651 to 28.801	0.329	6.029	−14.077 to 26.135	0.557
IQ	−1.049	−1.940 to −0.158	**0.021**	−1.032	−1.929 to −0.136	**0.024**	−0.958	−1.906 to −0.011	**0.047**
Variable	0.962	−0.660 to 2.584	0.245	5.234	−2.933 to 13.402	0.209	−0.560	−2.192 to 1.072	0.501
Variable × age	−0.128	−0.216 to −0.040	**0.004**	−0.437	−0.837 to −0.037	**0.032**	−0.033	−0.119 to 0.053	0.456
Variable × sex	−0.512	−2.581 to 1.557	0.628	−1.961	−12.432 to 8.509	0.714	0.901	−1.114 to 2.917	0.381
Variable × IQ	0.037	−0.069 to 0.143	0.495	−0.220	−0.724 to 0.285	0.393	0.040	−0.059 to 0.139	0.425
Stimulus type (1: smoking)	−0.949	−2.816 to 0.917	0.319	−0.962	−2.830 to 0.906	0.313	−1.095	−2.969 to 0.778	0.252
Variable × stimulus type	0.031	−0.170 to 0.232	0.764	0.232	−0.782 to 1.247	0.654	0.064	−0.123 to 0.252	0.503

*Note*: The predictor variable refers to either CPD, FTND, or QSU‐brief (smoking‐related variables). For the effects of smoking‐related variable (× stimulus type) on CE rates, one‐sided *P*‐values are reported, otherwise two‐sided. All regression model predictors, except for sex, were grand‐mean centred. None of the effects of smoking‐related variables (× stimulus type) or smoking‐related variables (× covariates) on the CE rate or Go‐RT were significant after Benjamini‐Hochberg correction. Significant *P*‐values are printed in bold.

Abbreviations: CE, commission error; CPD, cigarettes per day; FTND, Fagerström Test for Nicotine Dependence; Go‐RT, mean reaction time in Go trials; QSU‐brief, Questionnaire on Smoking Urges, brief version; IQ, intelligence quotient.

^a^ *n* = 119, because of missing values during data acquisition (see Appendix A.4.1). After data imputation, the results remained unchanged.

**FIGURE 1:**
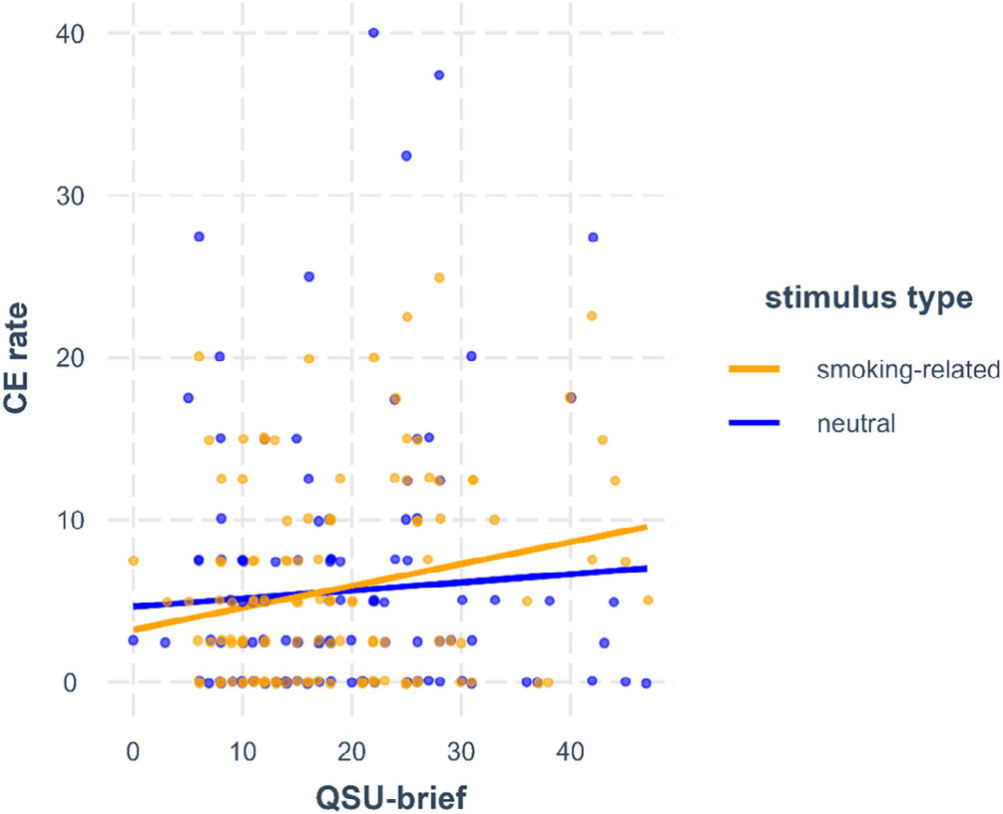
QSU‐brief score × stimulus type interaction on the CE rate in the smoking‐specific GNGT. For better interpretability, the QSU‐brief score was retained in its original scale (without grand‐mean centring). QSU‐brief, Questionnaire on Smoking Urges, brief version; CE, commission error.

Further, a significant interaction emerged between the QSU‐brief score and stimulus type on CE rates in the smoking‐specific GNGT (non‐significant after FDR‐correction). This effect indicates that higher craving was associated with a stronger tendency toward higher CE rates during smoking‐related compared to neutral trials (see Figure 1).

#### Effects of covariates on CE rates and Go‐RTs

In both GNGTs, higher age was associated with significantly lower CE rates and slower Go‐RTs. There was no clear evidence indicating a relationship between sex and CE rates or Go‐RTs. In the smoking‐specific GNGT, a higher IQ was associated with significantly faster Go‐RTs, whereas no association was observed with Go‐RTs in the general GNGT or CE rates.

#### Interactions between smoking‐related variables and covariates on CE rates and Go‐RTs

Because interaction analyses were not pre‐registered, they are considered exploratory. Furthermore, none of the interaction effects were significant after FDR‐correction (expect for the CPD × age effect on the CE rate in the general GNGT). Therefore, results should be regarded as preliminary and confirmed in subsequent studies before firm conclusions can be drawn.

Some significant interactions between age and smoking‐related variables on GNGT performance were observed (general GNGT: CE rate: CPD × age, QSU × age; smoking‐specific GNGT: CE rate: CPD × age, FTND × age; Go‐RT: CPD × age, FTND × age; for interaction plots see Appendix B, Figures B.1 and B.2). These effects indicate that, in older individuals (i.e. M_age_ + 1SD_age_ = 54 years), a greater number of CPD and a higher severity of tobacco dependence and craving were tendentially associated with higher CE rates and faster RTs in Go trials. Conversely, in younger individuals (i.e. M_age_ – 1SD_age_ = 28 years), a greater number of CPD and a higher severity of tobacco dependence and craving were tendentially associated with lower CE rates, but also slower Go‐RTs. Simply put, older individuals who smoked more, had greater tobacco dependence or reported stronger craving tended to respond faster in Go trials and tended to make more CEs by incorrectly responding in No‐Go trials. In contrast, younger individuals who smoked more, had greater tobacco dependence, or reported stronger craving tended to respond more slowly in Go trials and tended to make fewer CEs by correctly withholding responses in No‐Go trials. Overall, there was no clear evidence of significant interactions between smoking‐related variables and sex or IQ.

### Additional analyses

The main findings of the complementary analyses were: (1) no significant group differences in OE rates emerged (see Appendix C for results and Appendix E.1 [Appendix E] for a discussion); (2) results of regression models with years of smoking as predictor were largely consistent with results of the main analyses (see Appendix D for results and Appendix E.2 [Appendix E] for a discussion).

## DISCUSSION

The present study aimed to extend previous research regarding deficits in response inhibition within the context of smoking by comparing a large, age‐diverse smoking sample with healthy, non‐smoking controls, while considering individual differences in age, sex and intelligence [i.e. through the inclusion of those factors (covariates) and the examination of moderating effects]. Both general and smoking‐specific GNGTs were used. All measures exhibited good to excellent reliability, except for the FTND score (tobacco dependence severity), whose results should be interpreted with caution [37].

### Summary of findings

This study provides evidence that individuals who smoke demonstrate deficits in response inhibition compared to healthy, non‐smoking individuals. We hypothesised that the smoking group would show higher CE rates in the general GNGT compared to the non‐smoking group. This was confirmed by a significant group difference with medium effect size, which remained robust after accounting for individual differences in age, sex and IQ. Furthermore, Bayesian analyses provided substantial evidence for higher CE rates in the smoking group. These findings align with previous meta‐analytical research [4] (included empirical studies supporting the finding [10, 14]) and more recent empirical evidence [7, 13].

For the smoking‐specific GNGT, results are more complex. A simple comparison of groups (i.e. ANOVA; research question 1) did not yield a significant difference in the CE rate, nor did the regression model without considering age, sex and IQ differences in individuals. Importantly, after controlling for these variables, we observed a tendency (non‐significant after FDR‐correction) for higher CE rates in individuals who smoke (research question 2). This indicates that the manifestation of response inhibition deficits is not uniform, but rather varies depending on individual differences in age, sex and IQ. Their neglect may explain some of the non‐significant findings reported in previous studies [8, 10].

In terms of RTs, the smoking group responded significantly slower in Go trials of the smoking‐specific GNGT than the control group, with Bayesian analyses providing substantial support for a group difference. No such effect was observed on Go‐RTs in the general GNGT. These findings may indicate a generalised deficit in executive functioning in the smoking group [6], which became observable in the smoking‐specific GNGT. This is plausible, as the smoking‐specific GNGT likely required greater executive functioning (e.g. attentional control, working memory) because of the presentation of smoking‐related and neutral content compared to digits in the general GNGT.

In line with the IST and previous smoking‐specific GNGT studies [8, 12], the smoking group exhibited exacerbated responsivity toward smoking‐related stimuli, evidenced by shorter RTs in smoking‐related compared to neutral Go trials relative to the control group. In contrast, no significant group difference was found in CE rates during smoking‐related versus neutral trials. This contradicts the assumptions of the IST and previous studies reporting higher CE rates during smoking‐related trials in individuals who smoke [9, 12]. However, it aligns with the findings of Kräplin *et al*. [8], who only found group differences in Go‐RTs. One possible explanation is that the former studies used a contextual block design, whereas our study and Kräplin *et al*. used trial‐wise random presentation of smoking‐related and neutral stimuli. In interleaved designs, individuals may better compensate for deficits, whereas compensation is more difficult during smoking‐specific blocks. Alternatively, the overall low CE rates observed in the smoking‐specific GNGT (see Table 2) may reflect a floor effect, limiting the detection of significant group differences.

In research question 3, we hypothesised that higher scores on smoking‐related variables (i.e. CPD, tobacco dependence severity and craving) would be associated with greater deficits in response inhibition, particularly in response to smoking‐related stimuli. All findings should be interpreted with caution, as they became non‐significant after applying FDR‐correction. Higher scores in smoking‐related variables did not show a clear association with GNGT performance, with one exception: higher craving was associated with faster RTs in Go trials in the general GNGT, suggesting that the desire to smoke may accelerate reactions broadly, not just to smoking‐related stimuli. Additionally, higher craving was associated with higher CE rates toward smoking‐related compared to neutral stimuli in the smoking‐specific GNGT. This aligns with the IST, suggesting that craving may exacerbate response inhibition deficits, particularly toward smoking‐related stimuli.

In our exploratory examination of the moderating effects of age, sex and IQ within research question 3, age emerged as a potential moderator in the relationship between smoking‐related variables and GNGT performance. However, this effect was not consistently detectable and mostly not significant after FDR‐correction (see also Appendix E.2 for a discussion on the effect of smoking duration). Older (i.e. ≥ 54 years), heavier‐smoking individuals with greater severity of tobacco dependence and craving tended to show higher CE rates and faster RTs in Go trials. Conversely, younger individuals (i.e. ≤ 54 years) exhibited the tendency for a reversed pattern, with lower CE rates but slower Go‐RTs. Although the latter appears to contradict dual‐process models, the combination of slower Go‐RTs and reduced CE rates may reflect a strategic speed‐accuracy trade‐off to compensate for response inhibition deficits [6]. However, the moderating effect of age needs to be confirmed in studies with larger sample sizes, as our study was likely underpowered to detect interaction effects [38].

### Clinical and theoretical implications

Although our results support the significance of response inhibition in smoking, longitudinal studies are necessary to unravel their causal relationship. Moreover, ecological momentary assessment studies are required to better understand state and trait associations between response inhibition and smoking in everyday life. Clinically, our findings reinforce previous evidence that response inhibition training may effectively reduce smoking behaviour and craving [29]. Future research might investigate its efficacy in older, heavier‐smoking populations. Moreover, smoking‐specific inhibition training may be particularly effective in high‐risk situations, such as during periods of heightened craving.

### Limitations

Our results should be interpreted in light of several limitations. First, our covariates/moderators were not pre‐registered, which may raise concerns about *post hoc* adjustments, limiting credibility. However, age, sex and intelligence are common covariates in cognitive task data analyses. Second, the GNGTs used a long response window, resulting in lower task difficulty. This may have reduced sensitivity in differentiating response inhibition, potentially contributing to non‐significant findings. Third, exacerbated responsivity in individuals who smoke is likely not limited to smoking‐related stimuli, as previous research shows deficits toward other reward‐related stimuli, such as money [12]. Consequently, our findings should not be over interpreted as specific to smoking‐related stimuli. Fourth, our exclusion criteria for the non‐smoking sample did not account for passive smoking. However, research indicates that non‐smoking individuals passively exposed to smoking also exhibit executive functioning deficits [39]. Therefore, it remains unclear to what extent passive smoking may have affected response inhibition in the non‐smoking group. Lastly, we cannot exclude the possibility of sampling bias. This limitation arises from the lack of data on variables such as race or ethnicity. Additionally, participants were recruited for an intervention study aimed at reducing smoking behaviour, with a higher proportion of females and a predominance of highly educated individuals. In contrast, the smoking population in Germany is primarily male and less educated [40]. Therefore, the findings may not be generalisable to the general smoking population. However, we were able to recruit an older, more age‐diverse sample (age: 21–29: *n* = 29, 30–39: *n* = 30, 40–49: *n* = 25, 50–59: *n* = 27, 60–70: *n* = 11) with greater variation in smoking heaviness and/or duration compared to previous studies [8, 9, 10, 11, 17].

## CONCLUSION

The findings of the present study substantiate the notion that response inhibition plays a crucial role in smoking. Furthermore, our results suggest that the extent of deficits is person‐specific: greater deficits may be associated, first, with increased craving, second, during the exposure to smoking‐related cues and third, with heavier smoking and greater severity of tobacco dependence and craving in older individuals. In light of these findings, it would be interesting to investigate whether trainings aimed at improving response inhibition might exert positive effects on smoking behaviour, particularly among older individuals who smoke heavily or experiencing high levels of craving.

## AUTHOR CONTRIBUTIONS

**Franziska Motka**: Conceptualization (equal); data curation (equal); formal analysis (lead); investigation (equal); methodology (equal); project administration (equal); software (equal); validation (equal); visualization (lead); writing—original draft preparation (lead); writing—review and editing (equal). **Simone Kühn**: Conceptualization (equal); funding acquisition (lead); supervision (equal); writing—review and editing (equal). **Charlotte E. Wittekind**: Conceptualization (equal); data curation (equal); funding acquisition (equal); investigation (equal); methodology (equal); project administration (equal); software (equal); supervision (lead); validation (equal); writing—review and editing (equal).

## DECLARATION OF INTEREST

We have no known conflict of interest to disclose. The present study was supported by the European Union (ERC‐2016‐StG‐Self‐Control‐677804, ERC‐2022‐CoG‐BrainScape‐101086188) as part of a clinical trial. The clinical trial (German Clinical Trials Register, DRKS00014652; 23 April 2018), as well as the present study (AsPredicted.org, 172127, 24 April 2024), were pre‐registered.

## Supporting information


**Appendix A:** Additional methodological information
**Appendix A.1:** Participants and design
**Appendix A.1.1:** Flow of participants
**Appendix A.2:** Power analysis
**Appendix A.3:** Procedure and measures
**Appendix A.3.1:** Go/No‐Go Tasks and outcome measures
**Appendix A.4:** Statistical analysis
**Appendix A.4.1:** Data pre‐processing, aggregation, and reliability
**Appendix A.4.2:** Strategy of data analysis
**Appendix B:** Figures of interaction effects
**Appendix C:** Results with OE rates as outcome measure
**Appendix D:** Results with years of smoking as predictor
**Appendix E:** Supplementary discussion
**Appendix E.1:** OE rates
**Appendix E.2:** Years of smoking as predictor

## Data Availability

The data and final analysis code that support the findings is openly available in OSF at https://osf.io/rxu78/.

## Footnotes

1 In contrast, Detandt *et al*. [7] observed significantly reduced CE rates in individuals who smoke within smoking‐related compared to neutral trials.

2 Zhao and Chen [11] observed significantly higher CE rates only in trials with smoking‐social compared to neutral contexts, but found no significant difference between trials with smoking‐object versus neutral contexts.

3 Please note that Knott *et al*. [18] used the concept of gender rather than sex.

4 The AUDIT score was not included as a control variable in the regression models because, according to dual‐process models of addiction [1, 2], deficits in response inhibition—similar to the severity of tobacco dependence—are expected to be associated with higher alcohol consumption.

5 Robust explanatory effect size for *t* tests on trimmed means. Small effect: ξ = 0.10, medium effect: ξ = 0.30, see [36].

6 3 < BF_10_ < 10: indicates substantial evidence in favour of a difference between groups; 1 < BF_10_ < 3: indicates weak evidence in favour of a difference between groups; 1/3 < BF_10_ < 1: indicates weak evidence in favour of no difference between groups; 1/10 < BF_10_ < 1/3: indicates substantial evidence in favour of no difference between groups, see [34].
