# Supplementary material for: Deficits in general and smoking‐specific response inhibition in the Go/No‐Go task in individuals who smoke: A cross‐sectional analysis
Source: Addiction. 2025 Feb 19;120(7):1402–12. doi: 10.1111/add.70003 (PMC12128570; doi:10.1111/add.70003)
Supplement: Supplementary file 1 — Appendix A: Additional methodological information Appendix A.1: Participants and design Appendix A.1.1: Flow of participants Appendix A.2: Power analysis Appendix A.3: Procedure and measures Appendix A.3.1: Go/No‐Go Tasks and outcome measures Appendix A.4: Statistical analysis Appendix A.4.1: Data pre‐processing, aggregation, and reliability Appendix A.4.2: Strategy of data analysis Appendix B: Figures of interaction effects Appendix C: Results with OE rates as outcome measure Appendix D: Results with years of smoking as predictor Appendix E: Supplementary discussion Appendix E.1: OE rates Appendix E.2: Years of smoking as predictor [file ADD-120-1402-s001.docx]

**Deficits in general and smoking-specific response inhibition in the Go/No-Go task in individuals who smoke: A cross-sectional analysis**

*Supporting* *Information*

**Table of Contents**

[Appendix A: Additional methodological information 2](#_Toc184991324)

[Appendix A.1: Participants and design 2](#_Toc184991325)

[Appendix A.1.1: Flow of participants 3](#_Toc184991326)

[Appendix A.2: Power analysis 4](#_Toc184991327)

[Appendix A.3: Procedure and measures 4](#_Toc184991328)

[Appendix A.3.1: Go/No-Go tasks and outcome measures 8](#_Toc184991329)

[Appendix A.4: Statistical analysis 10](#_Toc184991330)

[Appendix A.4.1: Data pre-processing, aggregation, and reliability 10](#_Toc184991331)

[Appendix A.4.2: Strategy of data analysis 12](#_Toc184991332)

[Appendix B: Figures of interaction effects 15](#_Toc184991333)

[Appendix C: Results with OE rates as outcome measure 20](#_Toc184991334)

[Research question 1: Group difference analyses on OE rates: Smoking vs. control group 20](#_Toc184991335)

[Research question 2: Regression analyses with group and covariates as predictors 20](#_Toc184991336)

[Research question 3: Regression analyses with smoking-related variables and covariates in smoking individuals 21](#_Toc184991337)

[Effects of smoking-related variables and stimulus type on CE rates and Go-RTs 21](#_Toc184991338)

[Effects of covariates on CE rates and Go-RTs 21](#_Toc184991339)

[Interaction effects between smoking-related variables and covariates on CE rates and Go-RTs 22](#_Toc184991340)

[Appendix D: Results with years of smoking as predictor 24](#_Toc184991341)

[Research question 3: Regression analyses with smoking-related variables and covariates (including years of smoking) in smoking individuals 24](#_Toc184991342)

[Effects of smoking-related variables and stimulus type on CE rates and Go-RTs 24](#_Toc184991343)

[Effects of covariates on CE rates and Go-RTs 24](#_Toc184991344)

[Interaction effects between smoking-related variables and covariates on CE rates and Go-RTs 25](#_Toc184991345)

[Appendix E: Supplementary discussion 29](#_Toc184991346)

[Appendix E.1: OE rates 29](#_Toc184991347)

[Appendix E.2: Years of smoking as predictor 29](#_Toc184991348)

[References 31](#_Toc184991349)

# Appendix A: Additional methodological information

## Appendix A.1: Participants and design

Between May 2018 and October 2023^[[1]](#footnote-1)^, a total of 122 non-deprived individuals who smoke took part in a pre-registered intervention study on the efficacy of impulse control training as a stand-alone intervention in reducing tobacco consumption and craving (see German Clinical Trials Register, DRKS00014652; 23/04/2018), completed the baseline assessment and were included in the present cross-sectional study. Inclusion criteria were: (1) a total score of ≥ 3 in the Fagerström Test for Nicotine Dependence (FTND; [1]), (2) a carbon monoxide (CO) level in the exhaled air of ≥ 10 ppm, (3) age 18–70 years, (4) no use of nicotine replacement products (including e-cigarettes), (5) currently non-attendance of pharmacological or psychotherapeutic smoking cessation treatment, and (6) willingness to abstain from any therapy for smoking cessation during study participation. Exclusion criteria were: (1) moderate to severe substance use disorder other than tobacco within the last 12 months (i.e., 4 ≥ fulfilled criteria according to DSM-5 assessed with the Mini International Neuropsychiatric Interview [MINI; [2]]), (2) current or previous diagnosis of severe psychiatric (e.g., schizophrenia, bipolar disorder) or neurological (e.g., epilepsy, Parkinson disease, multiple sclerosis) disorders, (3) acute suicidality, (4) current pregnancy or nursing period, (5) uncorrectable vision, and (6) insufficient German language skills.

Between February 2024 and May 2024, a total of 69 age-matched healthy individuals with no history of smoking or any other substance use disorder were recruited as a control group. Inclusion criteria were: (1) age 18–70 years, and (2) ≤ 10 cigarettes smoked in lifetime. Exclusion criteria were: (1) current psychiatric disorders (assessed with the short structured clinical interview for diagnosing mental disorders according to DSM-5 and ICD-10 [Mini-DIPS-OA; [3,4]]), (2) previous diagnosis of severe psychiatric (e.g., schizophrenia, bipolar disorder, substance use disorder) or neurological (e.g., epilepsy, Parkinson disease, multiple sclerosis) disorders, (3) current intake of psychotropic medication, and (4) insufficient German language skills.

For the non-smoking group, the health-related rigorous criteria were selected based on the assumption that deficits in response inhibition represent a transdiagnostic risk factor in psychopathology [5]. Similar to previous studies in the field (e.g., [6–8]), our exclusion criteria for the non-smoking sample did not account for passive smoking. However, research has shown that individuals who have never smoked but have been passively exposed to smoking also exhibit deficits in executive functioning [9]. Accordingly, GNGT performance in the non-smoking group may have been affected by passive smoke exposure (see the limitations section in the main manuscript for a critical discussion).

We used convenience sampling (see the limitations section for a critical discussion), with participants from both groups recruited through various means, including notices in university buildings, ads on the university mailing list, flyers displayed in pharmacies and physicians’ waiting rooms, and social media platforms such as Facebook.

### Appendix A.1.1: Flow of participants

For the smoking group, *n* = 258 individuals were assessed for eligibility. A total of *n* = 136 were excluded due to the following reasons: 36 with FTND < 3 or no regular smoking; 31 did not show up for assessment; 28 used e-cigarettes, NRT, etc.; 21 had personal or logistic reasons (e.g., lack of time); 14 with probable substance dependence except tobacco; 3 with insufficient German language skills; 2 with severe neurological disorder; 1 with CO < 10; 1 with severe psychiatric disorder; 1 was aged < 18 or > 70 years; 1 with no reason documented.

For the control group, *n* = 108 individuals were assessed for eligibility. A total of *n* = 39 were excluded due to the following reasons: 17 with > 10 cigarettes smoked in lifetime; 9 had personal or logistic reasons (e.g., lack of time); 8 with current psychiatric disorders and/or intake of psychotropic medication; 2 with insufficient German language skills; 2 did not show up for assessment; 1 was aged < 18 or > 70 years.

## Appendix A.2: Power analysis

The sample size of *N* = 122 for the smoking group was determined in advance of the pre-registered clinical trial (see German Clinical Trials Register, DRKS00014652; 23/04/2018). The total sample size for the cross-sectional analysis was estimated to be *N* = 128 participants (i.e., 64 participants per group) to detect a medium effect of *d* = 0.5 by using G*Power [10], given *α* = .05 and *β* = .80 for two-sample *t*-tests (see the pre-registration of the current study: AsPredicted.org, #172127). For both groups, the recruitment stopped after the necessary sample size had been reached.

Although a previous meta-analysis revealed a small effect size for performance differences in the Go/No-Go task (GNGT) between smoking and control groups [11], a medium effect size was assumed for the current study. This is because, according to the IST, the use of smoking-related stimuli should result in increased deficits in response inhibition in individuals who smoke. Above, the a-priori estimated reliability of the GNGT measures for the smoking group exceeded a standard of good reliability (see Appendix A.4.1 and [12]; [13]). However, reliability is not fully inherent to a task [14], so this cannot be automatically conveyed to previous GNGT research. Therefore, poor reliability scores may have constrained the observed effect sizes in previous studies.

## Appendix A.3: Procedure and measures

All participants underwent an initial telephone screening (including an explanation of the study procedure, the clearance of in-/exclusion criteria, and the appointment arrangement). The assessment was conducted individually in a laboratory at the university. The fulfilment of the inclusion/exclusion criteria was verified at the beginning of the assessment. Following a sociodemographic interview, a series of questionnaires and experimental tasks were conducted. See Figure A.3.1 for an illustration of the assessment procedure. Some assessments specific to smoking (e.g., FTND) were not applicable to individuals in the control group. Consequently, the assessment duration was approximately 30 minutes shorter than in the smoking group. The participants received 8 Euros per hour for their participation in the assessment.

All questionnaires were administered in paper-pencil format, while the reaction time tasks were conducted using the computer-based software Inquisit (version 4, www.millisecond.com). The intervention study was designed to assess various potential working mechanisms of inhibitory control training. Accordingly, participants completed a series of tasks and questionnaires that were not relevant to the current investigation (see Figure A.3.1). In addition to the two GNGTs, the Stop-Signal task (SST; [15]) was administered in both a general and a smoking-specific version to assess response inhibition ability. However, in the course of publishing the results of the intervention study, split-half reliabilities were estimated, revealing that the outcome measure of the general SST in the smoking group was unsatisfactory (i.e., *r* = .472, *95% CI* [.316 – .628]; [12]). Meanwhile, the RTs of the smoking-specific SST could not be evaluated due to a programming error in the staircase procedure [12]. Consequently, the present investigation utilized solely data from both GNGTs to assess response inhibition.

**Figure A.3.1**

*Assessment procedure for smoking and control groups*

QSU

**Assessment of participants in the smoking group** (duration: ~ 2.5hr)

Drug and smoking history

Sociodemographic interview

WST

GNGT & SST^3^

IAT^1^

TAAS^1^

BIS-15^1^

GNGT & SST^3^

AAT^1^

MINI

AUDIT

CO value

FTND

**Assessment of participants in the control group** (duration: ~ 1.5hr)

Sociodemographic interview

Mini-DIPS

AUDIT

Drug and smoking history

IAT^1^

GNGT & SST^3^

GNGT & SST^3^

AAT^1^

WST

*Note*. FTND = Fagerström Test for Nicotine Dependence; CO = Carbon Monoxide; AUDIT = Alcohol Use Disorder Identification Test; MINI = Mini International Neuropsychiatric Interview for substance use disorders; QSU = Questionnaire of Smoking Urges, brief version; AAT = Approach-Avoidance task; WST = Wortschatztest; GNGT = Go/No-Go task; SST = Stop-Signal task; BIS-15 = Barratt Impulsiveness Scale, 15-item version; TAAS = Thoughts About Abstinence Scale; IAT = Implicit-Association Test; Mini-DIPS = Short structured clinical interview for diagnosing mental disorders according to DSM-5 and ICD-10.

^1^ not relevant for the current investigation.

^2^ both tasks (GNGT and SST) were given in both a general and smoking-specific version across two blocks. One block had both tasks in the general version and the other block had both tasks in the smoking-specific version. The order (block with general versions first vs. block with smoking-specific versions first) was randomized across participants. The two tasks in each block were absolved in a fixed order: first the GNGT, and second the SST.

### Appendix A.3.1: Go/No-Go tasks and outcome measures

Response inhibition was assessed by using both a general and smoking-specific GNGT. In the general GNGT, stimuli comprised white digits ranging from 1 to 8. Each digit was presented 40 times in a fully randomized sequence, resulting in 320 test trials. These were preceded by eighteen training trials. In each trial, a single digit was displayed centrally on a black screen. Participants were instructed to respond as quickly as possible to all digits except for the digits “3” and “6” by pressing the space bar with the index finger of their dominant hand (response window and stimulus presentation time: 1,000 ms). Given that each digit was presented with equal frequency, 75% of the trials required a response (Go trials; prepotent response), whereas in 25% of the trials, the response had to be withheld (No-Go trials). The inter-trial interval was 500 ms. If participants failed to respond correctly, the error message “error” was displayed for 400 ms.

The smoking-specific GNGT (adapted from 6) used the same set-up parameters (e.g., timings, trials). However, the stimuli consisted of 20 smoking-related and 20 neutral pictures. The pictures were selected from previous studies (e.g., [6,16,17]) and freely available online sources (see Figure A.3.1.1 for an impression of the stimuli).

**Figure A.3.1.1**

*Examples of smoking-related and neutral stimuli*


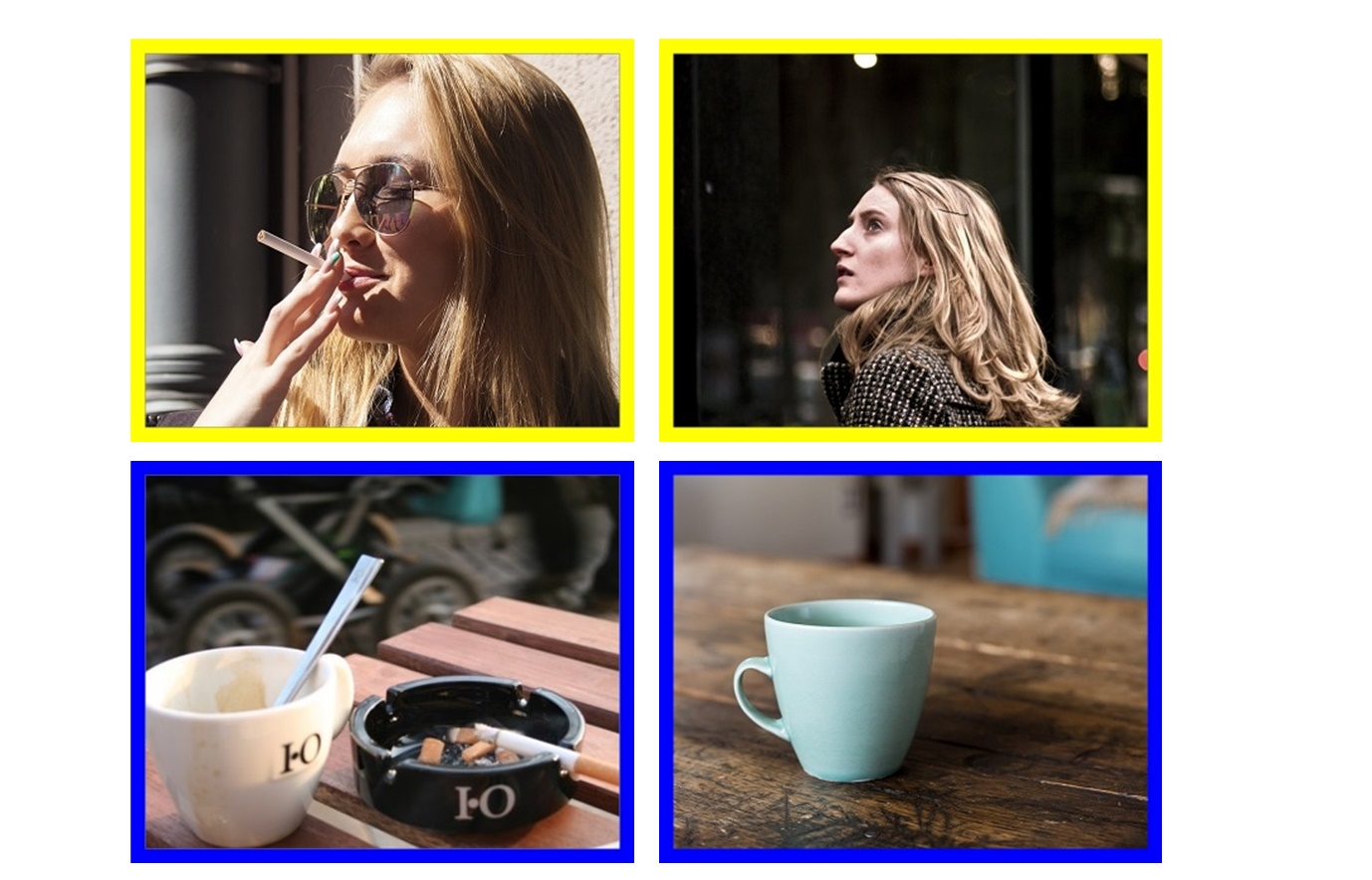


*Note*. Each smoking-related picture was paired with a neutral picture matched by content (e.g., a person versus a person smoking, coffee versus coffee and cigarettes). The colour frame (blue or yellow) signalled whether participants should respond or withhold a response.

Each of the 20 smoking-related pictures fell into one of 10 smoking-related categories, with two pictures per category (e.g., a person smoking, a person lighting a cigarette, coffee and cigarettes, alcoholic drink and cigarettes, etc.). Likewise, each of the 20 neutral pictures fell into one of 10 neutral categories, matched to the smoking-related categories (e.g., a person, a person brushing teeth, coffee, a glass of water, etc.). As a result, smoking-related pictures depicted content similar to that of neutral pictures (e.g., a person versus as person smoking, coffee versus coffee and cigarettes). This procedure ensured that the smoking-related and neutral pictures were comparable in complexity, although no formal statistical comparison was conducted.

In each trial, a single smoking-related or neutral stimulus framed in either blue or yellow was displayed centrally on a black screen. Participants were instructed to respond (i.e., Go or No-Go) based on the colour of the frame. The assignment of frame colour to response type (Go trials: yellow frame vs. Go trials: blue frame) was counterbalanced across participants. Each picture was presented eight times: six times (75%) as a Go and two times (25%) as a No-Go stimulus. The order of picture content (smoking-related versus neutral) and trial type (Go versus No-Go) was completely randomized. Unlike Luijten et al. [6], the present study utilized a longer stimulus presentation duration of 1,000 ms (compared to 200 ms). This should entail a more realistic and sustained exposure to smoking-related cues. Additionally, participants were not required to abstain from smoking before the experiment, unlike the one-hour abstinence requirement in Luijten et al. [6]. This approach should permit for a broader range of craving levels within the smoking group.

## Appendix A.4: Statistical analysis

### Appendix A.4.1: Data pre-processing, aggregation, and reliability

As pre-registered, participants with an OE rate above 35%^[[2]](#footnote-2)^ would have been excluded from further analyses on GNGT measures. As no participant reached this error rate, it can be concluded that task instruction adherence was excellent.

All questionnaires (see Table 1) were collected via paper-pencil format with the possibility that some questions might not have been answered. This resulted in missing data for the QSU-brief (*n* = 3) and AUDIT (*n* = 2). In the latter case, 16 participants received the 8-item version of the questionnaire instead of the 10-item version, which resulted in a higher rate of missing data. It was assumed that the missing items in the questionnaires were missing at random, as participants completed them individually without expecting negative or positive consequences for their answers. For transparency, the final sample sizes for both variables (QSU-brief: *n* = 119; AUDIT: *n* = 104) are indicated in Tables 1, 4 and 5.

Missing data was handled as follows. Since the AUDIT score was used solely to describe the sample characteristics, missing values on this measure were not imputed. In the models using the QSU-brief score as a predictor in research question 3, the sample size was reduced (*n* = 119, instead of *n* = 122). The rate of missing data was 3 out of 122 (~2.46%). Some researchers suggest a missing data rate below 5% is neglectable (e.g., [19]). However, we also examined the robustness of our findings after imputing missing values. For two participants, only one of the 10 items on the QSU-brief was missing. These values were replaced by the participant’s mean score on the remaining QSU-brief items. In the third case, the QSU-brief score was missing because the examiner accidentally omitted the questionnaire. This missing score was replaced by the sample mean QSU-brief score. After this imputation procedure, the results for all models using the QSU-brief score as a predictor in research question 3 remained unchanged.

To estimate the Spearman-Brown corrected split-half reliabilities of the GNGT measures, the R package *splithalfr* [20] was used with 5,000 random splits (see Table A.4.1.1). For questionnaire data, Cronbach’s alpha was calculated (smoking group: QSU-brief: *α* = .805, *95% CI* [.738; .851]; FTND: *α* = .481, *95% CI* [.334; 587]).

**Table A.4.1.1**

*Split-half reliability scores of task measures*

| Measures | Smoking group | |  | Control group | |
| --- | --- | --- | --- | --- | --- |
|  | *r* | *95%* *CI* |  | *r* | *95%* *CI* |
| **General GNGT** |  |  |  |  |  |
| CE rate | .858 | .802 – .914 |  | .817 | .702 – .933 |
| Go-RT | .986 | .983 – .990 |  | .982 | .976 – .987 |
| **Smoking-specific GNGT** |  |  |  |  |  |
| CE rate (all trials) | .838 | .773 – .902 |  | .791 | .655 – .928 |
| CE rate (smoking-related trials) | .613 | .529 – .696 |  | .609 | .417 – .800 |
| CE rate (neutral trials) | .773 | .678 – .867 |  | .646 | .422 – .871 |
| Go-RT (all trials) | .993 | .991 – .995 |  | .987 | .982 – .993 |
| Go-RT (smoking-related trials) | .986 | .982 – .990 |  | .975 | .966 – .985 |
| Go-RT (neutral trials) | .986 | .982 – .989 |  | .975 | .963 – .986 |

*Note*. *r* = Split-half reliability, Spearman-Brown corrected; CI = Confidence Interval; GNGT = Go/No-Go task; CE = Commission Error; Go-RT = mean Reaction Time in Go trials.

### Appendix A.4.2: Strategy of data analysis

In research question 1, we used *t*-tests and ANOVAs without considering covariates for comparative purposes, as these statistical methods have been predominantly employed in previous GNGT studies (e.g., [6,8,21–23]). For the statistical analyses addressing research questions 2 and 3, we chose linear regression models because they provide advantages over ANOVAs and *t*-tests by accommodating the modelling of nested structures (i.e., performance on smoking-related and neutral trials within each individual) and offering greater flexibility in incorporating covariates [24].

The assumptions for each statistical model were tested. For all models, we identified influential data points (i.e., outliers and/or leverage points, assessed using boxplots, studentized residuals, leverage plots, and Cook’s distance). Additionally, we found that some model data violated the assumptions of normality (of residuals) and/or homogeneity of variances. Given that influential data points were primary concerns in our statistical analyses, we decided to employ data trimming for research question 1 and robust regression techniques for research questions 2 and 3. These methods mitigate the impact of influential data points while also addressing issues related to non-normality and non-homogeneity of variances. This approach aligns with the general recommendations of Field and Wilcox [25] on handling the common challenges posed by violated assumptions in experimental data, which can easily distort the results obtained through standard statistical methods.

For research question 1 regarding the general GNGT, robust two-sample *t*-tests on trimmed means (*yuenbt* function, *WRS2* package, version 1.1-4; 26; with the default trim proportion of 0.2 and 5,000 bootstraps) were conducted to examine the differences in performance measures between the smoking and control groups. Tests on trimmed means offer a good solution for skewed distributions, providing a balance between utilizing the mean (no trimming) and the median (maximum amount of trimming; [27]). The effect size is provided by *ξ*, a robust explanatory measure with values of = .10, .30, and .50 corresponding to a small, medium, and large effect [26]. For research question 1 regarding the smoking-specific GNGT, robust two-way mixed measures ANOVAs on trimmed means (*bwtrim* function, *WRS2* package, with the default trim proportion of 0.2) were performed, with the between-subject factor group (smoking/non-smoking) and the within-subject factor stimulus type (smoking-related/neutral). As the *bwtrim* function does not provide effect sizes, *ξ* was estimated with the *yuen* (independent samples) and *yuend* (dependent samples) functions. The classical frequentist analyses on group effects were complemented by post-hoc Bayesian two-sample *t*-tests (*ttestBF* function, *BayesFactor* package; [28]). Bayes factors (BF_10_) > 3 were interpreted as substantial evidence in favour of a difference between groups [29].

For research question 2 regarding the general GNGT, robust multiple linear regressions (*rlm* function, *MASS* package, version 7.3-58.4; [30]) were conducted to examine whether group (smoking/non-smoking) was associated with GNGT performance while controlling for age, sex, and IQ. Data of the smoking-specific GNGT had a 2-level structure, with smoking-related and neutral trials nested within each subject. Therefore, robust linear mixed-effects models were utilized (*rlmer* function, *robustlmm* package, version 3.3-1; [31]) to examine whether group (smoking/non-smoking), stimulus type (smoking-related/neutral), and their interaction were associated with the performance in the smoking-specific GNGT while controlling for age, sex, and IQ.

For research question 3, the performance of the smoking group in the general GNGT was examined by conducting three robust multiple linear regression models (*rlm* function) with either CPD, the FTND, or QSU-brief score (smoking-related variables) as main predictor. Each model included age, sex, and IQ as control variables as well as their interactions with the respective main predictor. For the performance in the smoking-specific GNGT, again, robust linear mixed-effects models were utilized, including stimulus type (smoking-related/neutral) and its interaction with CPD, FTND, or QSU-brief as additional predictors.

The Benjamini-Hochberg correction [32] was employed to adjust for several effects of interest on each GNGT outcome measure within each research question. The method controls the false discovery rate at 5% and demonstrates good performance with substantial power and reasonable protection against the Type I error [33,34]. For example, in each of research questions 1 and 2, three effects were corrected for the CE rate: the effect of group in the general GNGT as well as the effects of group and group × stimulus type in the smoking-specific GNGT. In research question 3, nine effects were corrected for the CE rate: the effects of the three smoking-related variables on the CE rate in the general GNGT, as well as the effects of the three smoking-related variables and smoking-related variables × stimulus type on the CE rate in the smoking-specific GNGT. The exploratory investigated interaction effects (smoking-related variables × age, × sex, and × IQ) were FDR-corrected separately (i.e., 18 effects for each GNGT outcome measure). All regression model predictors, except for sex, were grand-mean centred.

# Appendix B: Figures of interaction effects

To the best of our knowledge, there is no package providing interaction plots for the robust regression model functions used for our analyses (see Appendix A.4.2). Therefore, the plots were compiled using the *interactions* package [35] on conventional (non-robust) regression models (*lm* and *lmer* functions, *lme4* package, version 1.1-34; [36]). The direction of the effects observed in the conventional and robust models remained consistent.

**Figure B.1**

*Smoking-related variable×age interaction plots on CE rates, Go-RTs, and OE rates in the general GNGT*


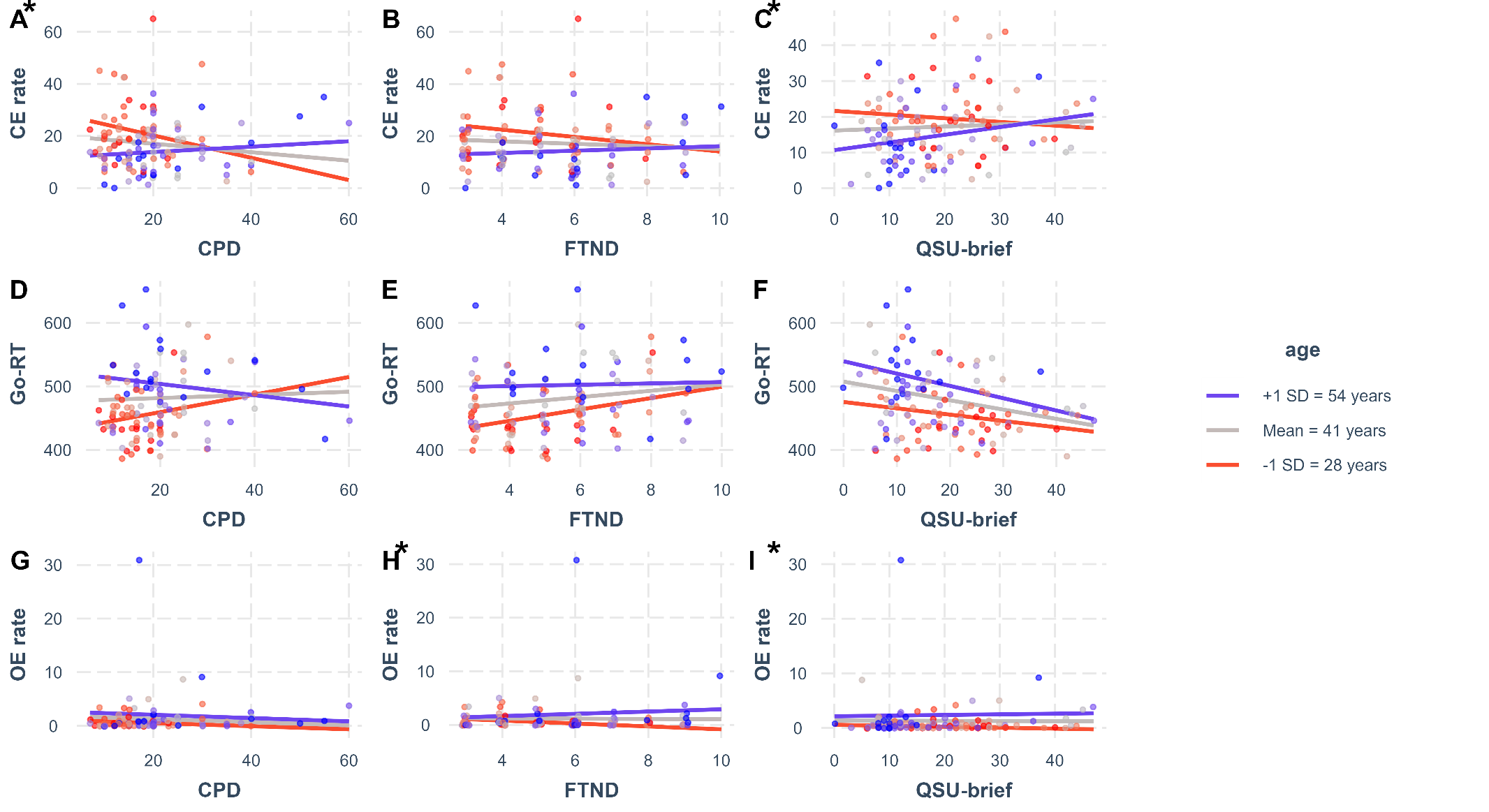


*Note*. Significant smoking-related variable*×*age interactions are indicated by *. For better interpretability, variables were retained in their original scale (without grand-mean centering). CPD = Cigarettes Per Day; FTND = Fagerström Test for Nicotine Dependence; QSU-brief = Questionnaire on Smoking Urges, brief version; CE = Commission Error; OE = Omission Error; Go-RT = mean Reaction Time in Go trials.

**Figure B.2**

*Smoking-related variable×age interaction plots on CE rates, Go-RTs, and OE rates in the smoking-specific GNGT*


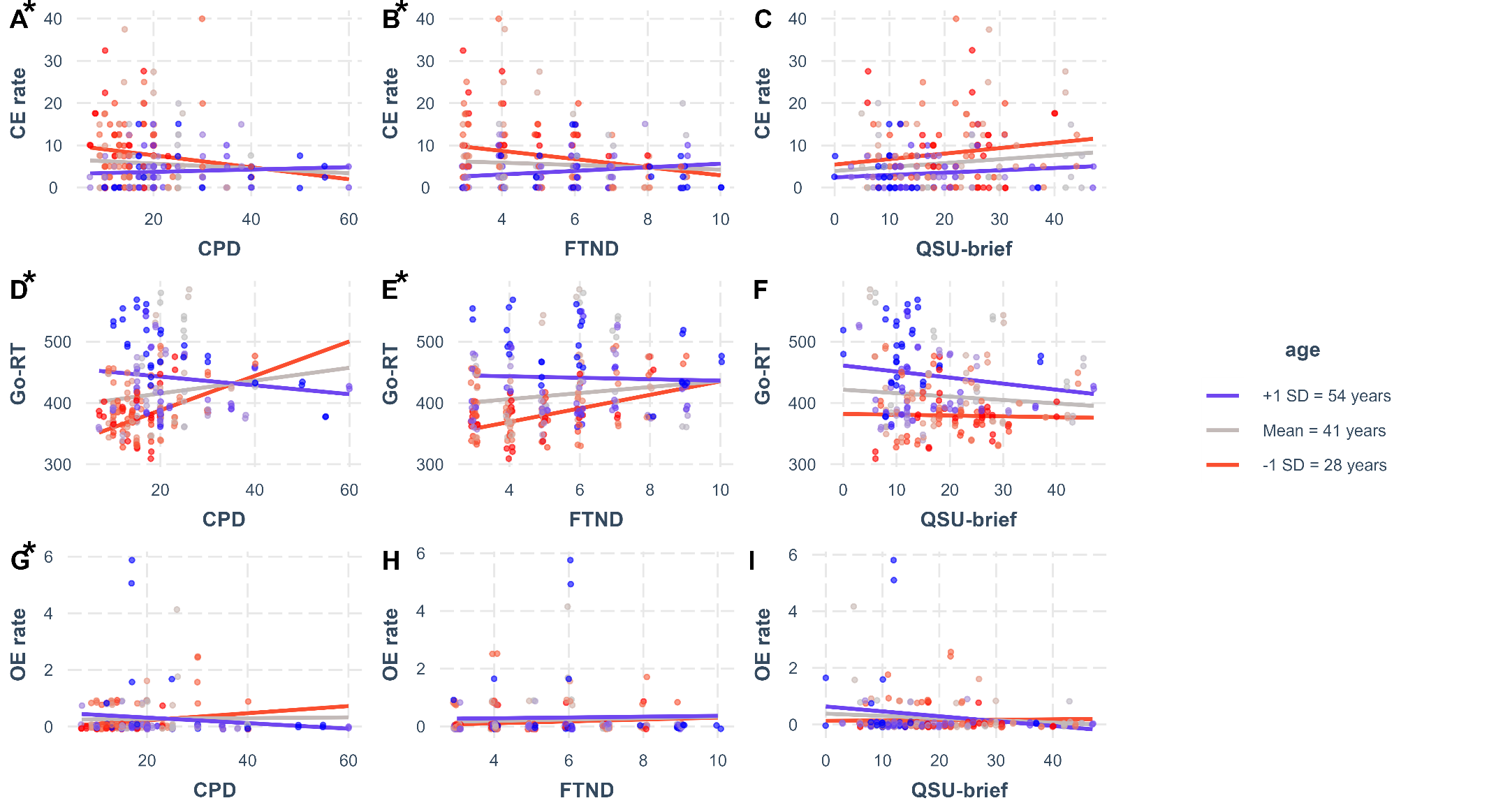


*Note*. Significant smoking-related variable*×*age interactions are indicated by *. For better interpretability, variables were retained in their original scale (without grand-mean centering). CPD = Cigarettes Per Day; FTND = Fagerström Test for Nicotine Dependence; QSU-brief = Questionnaire on Smoking Urges, brief version; CE = Commission Error; OE = Omission Error; Go-RT = mean Reaction Time in Go trials.

# Appendix C: Results with OE rates as outcome measure

Both GNGTs were implemented with a relatively large response window of 1,000 ms, which resulted in overall low OE rates (see Table 2). Accordingly, the OE rate may not be a sensitive measure for differentiating between individuals’ performance. However, readers may be interested in the results of OE rates as outcome measure, and thus, we have provided them here. A summary of the results is given in the Results section of the main manuscript.

## Research question 1: Group difference analyses on OE rates: Smoking vs. control group

Given the minimal overall variability in OE rates, trimming was not employed in their statistical analyses. Neither in the general GNGT (*T_y_* = -1.04, *P*-value = .293, *95%* *CI* [-0.37; 0.11], *ξ* = .14^[[3]](#footnote-3)^, BF_10_ = 0.334^[[4]](#footnote-4)^), nor in the smoking-specific GNGT (*Q*[1, 119.81] = 0.27, *P*-value = .604, *ξ* = .07, BF_10_ = 0.18), did frequentist and Bayes statistics provide evidence for a difference between groups on OE rates. In the smoking-specific GNGT, the main effects of stimulus type lacked statistical significance, *Q*(1, 120.80) = 0.43, *P*-value = .511, *ξ* = .03. Also, there was no statistical support for a significant interaction between group and stimulus type, *Q*(1, 120.80) < 0.01, *P*-value = .968.

## Research question 2: Regression analyses with group and covariates as predictors

Regression models did not yield substantial evidence to suggest a difference in OE rates between the smoking and control groups in either GNGT (see Table C.1). Unlike the models using CE rates and Go-RTs as the outcome measure, no significant association between age and OE rates was observed. Also, no significant association with sex and IQ emerged.

**Table C.1**

*Results of regression models on OE rates in both GNGTs*

|  | **OE rate** | | |
| --- | --- | --- | --- |
| Predictors | *β* | *95% CI* | *P*-value |
|  | **General GNGT** | | |
| Intercept | 0.418 | 0.222 – 0.614 | **<.001** |
| Group (1: smoking) | 0.092 | -0.098 – 0.282 | .341 |
| Age | 0.006 | -0.001 – 0.014 | .084 |
| Sex (1: female) | -0.032 | -0.217 – 0.153 | .733 |
| IQ | -0.004 | -0.013 – 0.005 | .376 |
|  | **Smoking-specific GNGT**^1^ | | |
| Intercept | 0.336 | 0.150 – 0.522 | **<.001** |
| Group (1: smoking) | -0.111 | -0.312 – 0.091 | .281 |
| Age | 0.001 | -0.005 – 0.006 | .758 |
| Sex (1: female) | -0.027 | -0.168 – 0.113 | .703 |
| IQ | -0.003 | -0.010 – 0.004 | .379 |
| Stimulus type (1: smoking) | -0.127 | -0.352 – 0.097 | .266 |
| Stimulus type × Group | 0.125 | -0.156 – 0.407 | .383 |

*Note*. All regression model predictors, except for sex, were grand-mean centered. CI = Confidence Interval; GNGT = Go/No-Go task.

^1^ coefficients multiplied by 10^13^ for readability.

## Research question 3: Regression analyses with smoking-related variables and covariates in smoking individuals

### Effects of smoking-related variables and stimulus type on CE rates and Go-RTs

Smoking-related variables (i.e., CPD, FTND, and QSU-brief) did not show conclusive evidence of a relationship with performance in the GNGTs. Also, no significant interactions were observed between smoking-related variables and stimulus type on GNGT performance.

### Effects of covariates on CE rates and Go-RTs

Contrary to the regression models that investigated CE rates and Go-RTs as outcome measures, age was not consistently associated with higher OE rates (see Table C.2; only in the general GNGT, exclusively in the model that included the QSU-brief score as main predictor). Also, a higher IQ was not consistently related to lower OE rates in the smoking-specific GNGT (only in the model with the QSU-brief score as main predictor). However, in line with models that investigated CE rates and Go-RTs, IQ was not significantly associated with Go-RTs in the general GNGT or CE rates.

### Interaction effects between smoking-related variables and covariates on CE rates and Go-RTs

None of the interaction effects were significant after FDR-correction; therefore, results should be regarded as preliminary and confirmed in subsequent studies before firm conclusions can be drawn. Some significant interactions between age and smoking-related variables on the performance in the GNGTs were observed (general GNGT: FTND×age, QSU-brief×age; smoking-specific GNGT: CPD×age; for interaction plots see Appendix B, Figures B.1 and B.2). These effects indicate that older individuals (i.e., *M*_age_+1 *SD*_age_ = 54 years) with higher severity of tobacco dependence and craving tended to exhibit higher OE rates in the general GNGT. Conversely, younger individuals (i.e., *M*_age_-1 *SD*_age_ = 28 years) with higher severity of tobacco dependence and craving tended to exhibit lower OE rates. Simply put, older individuals who had greater tobacco dependence or reported stronger craving tended to make more OEs by incorrectly non-responding in Go trials. In contrast, younger individuals who had greater tobacco dependence or reported stronger craving tended to make fewer OEs by correctly responding in Go trials. In the smoking-specific GNGT, older individuals with a higher number of CPD tended to show lower OE rates, while in younger individuals, a higher number of CPD was tendentially associated with higher OE rates. Simply put, older individuals who smoked more tended to make fewer OEs by correctly non-responding in Go trials. In contrast, younger individuals who smoked more tended to make more OEs by incorrectly responding in Go trials. Again, **there was no clear evidence of significant interactions** between smoking-related variables and sex or IQ.

**Table C.2**

*Results of regression models on OE rates in both GNGTs*

| Predictors | CPD | | | FTND | | | QSU-brief | | |
| --- | --- | --- | --- | --- | --- | --- | --- | --- | --- |
|  | *β* | *95% CI* | *P-*value | *β* | *95% CI* | *P-*value | *β* | *95% CI* | *P-*value |
|  | **OE rate** | | | | | | | | |
|  | **General GNGT** | | | | | | | | |
| Intercept | 0.505 | 0.324 – 0.686 | **<.001** | 0.513 | 0.326 – 0.700 | **<.001** | 0.512 | 0.327 – 0.698 | **<.001** |
| Age | 0.007 | -0.003 – 0.017 | .149 | 0.008 | -0.002 – 0.018 | .100 | 0.017 | 0.006 – 0.027 | **.002** |
| Sex (1: female) | -0.053 | -0.288 – 0.182 | .657 | -0.056 | -0.301 – 0.189 | .651 | 0.051 | -0.198 – 0.299 | .687 |
| IQ | -0.007 | -0.018 – 0.004 | .214 | -0.009 | -0.020 – 0.003 | .139 | -0.001 | -0.013 – 0.010 | .827 |
| Variable | 0.007 | -0.012 – 0.027 | .467 | -0.019 | -0.123 – 0.085 | .715 | 0.020 | -0.00004 – 0.040 | .050 |
| Variable × Age | 0.0004 | -0.001 – 0.001 | .462 | 0.006 | 0.001 – 0.011 | **.029** | 0.001 | 0.0004 – 0.003 | **.008** |
| Variable × Sex | -0.0003 | -0.025 – 0.025 | .980 | 0.064 | -0.070 – 0.197 | .345 | -0.006 | -0.031 – 0.019 | .638 |
| Variable × IQ | 0.001 | -0.0002 – 0.002 | .105 | 0.002 | -0.004 – 0.009 | .462 | -0.0002 | -0.001 – 0.001 | .775 |
|  | **Smoking-specific GNGT**^1^ | | | | | | | | |
| Intercept | 0.240 | 0.095 – 0.385 | **.001** | 0.264 | 0.087 – 0.442 | **.003** | 0.049 | 0.017 – 0.081 | **.003** |
| Age | -0.004 | -0.011 – 0.003 | .247 | -0.002 | -0.010 – 0.006 | .655 | -0.0002 | -0.002 – 0.001 | .753 |
| Sex (1: female) | 0.023 | -0.136 – 0.183 | .773 | 0.017 | -0.179 – 0.214 | .862 | 0.0003 | -0.036 – 0.036 | .987 |
| IQ | -0.008 | -0.015 – -0.0003 | **.040** | -0.009 | -0.018 – 0.0006 | .068 | -0.002 | -0.004 – -0.0004 | **.018** |
| Variable | 0.011 | -0.005 – 0.027 | .164 | 0.083 | -0.015 – 0.181 | .097 | -0.001 | -0.005 – 0.002 | .460 |
| Variable × Age | -0.001 | -0.001 – -0.00003 | **.042** | -0.003 | -0.007 – 0.001 | .175 | -0.00002 | -0.0002 – 0.0001 | .760 |
| Variable × Sex | 0.004 | -0.013 – 0.021 | .651 | -0.066 | -0.172 – 0.041 | .229 | 0.001 | -0.002 – 0.005 | .505 |
| Variable × IQ | -0.0004 | -0.001 – 0.0004 | .322 | -0.007 | -0.012 – -0.002 | **.007** | 0.0002 | -0.000006 – 0.0003 | .058 |
| Stimulus type (1: smoking) | -0.018 | -0.173 – 0.137 | .824 | -0.010 | -0.201 – 0.182 | .922 | -0.001 | -0.035 – 0.033 | .954 |
| Variable × Stimulus type | -0.009 | -0.026 – 0.008 | .287 | -0.083 | -0.187 – 0.021 | .116 | -0.002 | -0.005 – 0.002 | .308 |

*Note*. The predictor *Variable* refers to either CPD, FTND, or QSU. All regression model predictors, except for sex, were grand-mean centered. None of the effects of smoking-related variables(×stimulus type) on the CE rate or Go-RT were significant after Benjamini-Hochberg correction. CPD = Cigarettes Per Day; FTND = Fagerström Test for Nicotine Dependence; QSU-brief = Questionnaire on Smoking Urges, brief version; CI = Confidence Interval; GNGT = Go/No-Go task. ^1^ coefficients multiplied by 10^13^ for readability.

# Appendix D: Results with years of smoking as predictor

Within research question 3, we aimed to control for the effect of age, but were also interested in the effect of smoking duration (in years) on the GNGT performance. However, it was not possible to include both variables in the regression models due to their multicollinearity (observed correlation between age and years of smoking: *r* = .909). Therefore, regression models were performed separately with age and years of smoking as predictors. A summary of the results is given in the Results section of the main manuscript. The discussion of the results is provided in Appendix E.2.

## Research question 3: Regression analyses with smoking-related variables and covariates (including years of smoking) in smoking individuals

### Effects of smoking-related variables and stimulus type on CE rates and Go-RTs

As in regression models with age as predictor, smoking-related variables (i.e., CPD, FTND, and QSU-brief) did not show conclusive evidence of a relationship with performance in the GNGTs (see Tables D.1 and D.2), with one exception: higher QSU-brief scores were tendentially associated with faster Go-RTs in the general GNGT (non-significant after FDR-correction). Once more, in the smoking-specific GNGT, a significant interaction was observed between the QSU-brief score and stimulus type. This effect indicates that higher reported craving was associated higher CE rates during smoking-related trials compared to neutral trials.

### Effects of covariates on CE rates and Go-RTs

In both GNGTs, a longer history of smoking was associated with significantly lower CE rates and slower Go-RTs. There was no clear evidence indicating an association between sex and CE rates or Go-RTs. In the smoking-specific GNGT, a higher IQ was associated with significantly faster Go-RTs (although this effect was non-significant in the model with QSU-brief as main predictor), whereas IQ was not significantly related to Go-RTs in the general GNGT or CE rates.

### Interaction effects between smoking-related variables and covariates on CE rates and Go-RTs

Since the interaction effects between smoking-related variables and age, sex, and IQ were not pre-registered, they should be regarded as exploratory. Furthermore, none of the interaction effects were significant after FDR-correction; therefore, results should be regarded as preliminary and confirmed in subsequent studies before firm conclusions can be drawn.

Again, regression models revealed some significant interaction effects between years of smoking and smoking-related variables (i.e., CPD, FTND, and QSU-brief) on the performance in the GNGTs. In contrast to the models with age as predictor, the interactions between years of smoking and the FTND and QSU-brief score on CE rates in the general GNGT did not provide conclusive evidence of a relationship. Meanwhile, a significant interaction between years of smoking and CPD on Go-RTs in the general GNGT was observed. These effects indicate that in individuals with a longer history of smoking (i.e., *M*_years of smoking_+1 *SD*_years of smoking_ = 35 years of smoking), a greater number of CPD and a higher severity of tobacco dependence were tendentially associated with higher CE rates and faster RTs in Go trials. Conversely, in individuals with a shorter history of smoking (i.e., i.e., *M*_years of smoking_-1 *SD*_years of smoking_ = 9 years of smoking), a greater number of CPD and a higher severity of tobacco dependence were tendentially associated with lower CE rates, but also slower Go-RTs. Simply put, individuals with a longer history of smoking, who smoked more or reported stronger craving, tended to respond faster in Go trials and tended to make more CEs by incorrectly responding in No-Go trials. In contrast, individuals with a shorter history of smoking, who smoked more or reported stronger craving, tended to respond more slowly in Go trials and tended to make fewer CEs by correctly withholding responses in No-Go trials. Again, **there was no clear evidence of significant interactions** between smoking-related variables and sex or IQ.

**Table D.1**

*Results of the regression models on CE rates and Go-RTs in the general GNGT with years of smoking as predictor*

| Predictor | CPD | | | FTND | | | | | QSU | | |
| --- | --- | --- | --- | --- | --- | --- | --- | --- | --- | --- | --- |
|  | *β* | *95% CI* | *P-*value | | *β* | *95% CI* | *P-*value | *β* | | *95% CI* | *P-*value |
|  | **CE rate** | | | | | | | | | | |
| Intercept | 15.786 | 13.231 – 18.340 | **<.001** | | 15.980 | 13.232 – 18.728 | **<.001** | 16.048 | | 13.329 – 18.766 | **<.001** |
| Years of smoking | -0.198 | -0.335 – -0.062 | **.005^*^** | | -0.197 | -0.344 – -0.050 | **.009^*^** | -0.158 | | -0.309 – -0.008 | **.040** |
| Sex (1: female) | -0.055 | -3.378 – 3.267 | .974 | | -0.175 | -3.726 – 3.376 | .922 | 0.114 | | -3.536 – 3.765 | .951 |
| IQ | -0.094 | -0.248 – 0.059 | .225 | | -0.096 | -0.263 – 0.070 | .253 | -0.032 | | -0.205 – 0.141 | .715 |
| Variable | -0.135 | -0.420 – 0.150 | .825 | | -0.149 | -1.685 – 1.387 | .576 | -0.002 | | -0.300 – 0.295 | .506 |
| Variable × Years of smoking | 0.020 | 0.006 – 0.034 | **.005** | | 0.058 | -0.015 – 0.131 | .118 | 0.011 | | -0.005 – 0.027 | .176 |
| Variable × Sex | 0.313 | -0.044 – 0.670 | .085 | | 0.431 | -1.512 – 2.374 | .661 | 0.041 | | -0.333 – 0.415 | .830 |
| Variable × IQ | -0.001 | -0.020 – 0.017 | .876 | | -0.003 | -0.096 – 0.090 | .948 | -0.010 | | -0.028 – 0.009 | .292 |
|  | **Go-RT** | | | | | | | | | | |
| Intercept | 478.994 | 465.755 – 492.234 | **<.001** | | 477.618 | 463.750 – 491.487 | **<.001** | 475.965 | | 462.431 – 489.498 | **<.001** |
| Years of smoking | 1.616 | 0.910 – 2.322 | **<.001^*^** | | 1.530 | 0.787 – 2.273 | **<.001^*^** | 1.574 | | 0.824 – 2.323 | **<.001^*^** |
| Sex (1: female) | -11.909 | -29.125 – 5.308 | .173 | | -10.993 | -28.914 – 6.929 | .227 | -10.992 | | -29.162 – 7.178 | .233 |
| IQ | -0.402 | -1.196 – 0.392 | .318 | | -0.516 | -1.354 – 0.323 | .226 | -0.703 | | -1.563 – 0.157 | .108 |
| Variable | 0.097 | -1.381 – 1.575 | .897 | | 3.638 | -4.114 – 11.390 | .354 | -1.491 | | -2.972 – -0.010 | **.048** |
| Variable × Years of smoking | -0.078 | -0.150 – -0.006 | **.034** | | -0.244 | -0.611 – 0.123 | .190 | -0.047 | | -0.128 – 0.033 | .246 |
| Variable × Sex | -0.171 | -2.021 – 1.680 | .855 | | -1.409 | -11.215 – 8.396 | .776 | 1.479 | | -0.382 – 3.340 | .118 |
| Variable × IQ | 0.087 | -0.008 – 0.182 | .072 | | -0.203 | -0.672 – 0.267 | .395 | 0.062 | | -0.031 – 0.154 | .189 |

*Note*. The predictor *Variable* refers to either CPD, FTND, or QSU. For the effects of smoking-related variables on CE rates, one-sided *p*-values are reported, otherwise two-sided. All regression model predictors, except for sex, were grand-mean centered. CPD = Cigarettes Per Day; FTND = Fagerström Test for Nicotine Dependence; QSU = Questionnaire on Smoking Urges, brief version; CI = Confidence Interval; CE = Commission Error; Go-RT = mean Reaction Time in Go trials. ^*^ significant after Benjamini-Hochberg correction.

**Table D.2**

*Results of the regression models on CE rates and Go-RTs in the smoking-specific GNGT with years of smoking as predictor*

| Predictor | CPD | | | FTND | | | QSU | | |
| --- | --- | --- | --- | --- | --- | --- | --- | --- | --- |
|  | *β* | *95% CI* | *P-*value | *β* | *95% CI* | *P-*value | *β* | *95% CI* | *P-*value |
|  | **CE rate** | | | | | | | | |
| Intercept | 4.442 | 3.055 – 5.828 | **<.001** | 4.313 | 2.916 – 5.711 | **<.001** | 4.635 | 3.222 – 6.048 | **<.001** |
| Years of smoking | -0.086 | -0.155 – -0.016 | **.016^*^** | -0.101 | -0.172 – -0.030 | **.005^*^** | -0.098 | -0.172 – -0.024 | **.009^*^** |
| Sex (1: female) | 0.510 | -1.185 – 2.205 | .555 | 0.647 | -1.056 – 2.350 | .457 | 0.131 | -1.664 – 1.925 | .887 |
| IQ | -0.036 | -0.114 – 0.042 | .370 | -0.034 | -0.113 – 0.046 | .409 | -0.032 | -0.117 – 0.053 | .455 |
| Variable | -0.072 | -0.226 – 0.083 | .819 | -0.097 | -0.876 – 0.682 | .596 | 0.006 | -0.147 – 0.159 | .530 |
| Variable × Years of smoking | 0.008 | 0.001 – 0.015 | **.029** | 0.043 | 0.008 – 0.078 | **.015** | -0.004 | -0.012 – 0.004 | .290 |
| Variable × Sex | 0.026 | -0.156 – 0.208 | .780 | -0.519 | -1.450 – 0.413 | .275 | -0.059 | -0.243 – 0.124 | .526 |
| Variable × IQ | -0.008 | -0.017 – 0.002 | .105 | -0.024 | -0.069 – 0.020 | .283 | -0.004 | -0.013 – 0.005 | .432 |
| Stimulus type (1: smoking) | 0.527 | -0.418 – 1.471 | .275 | 0.507 | -0.424 – 1.439 | .286 | 0.441 | -0.476 – 1.359 | .346 |
| Variable × Stimulus type | 0.002 | -0.099 – 0.104 | .484 | 0.033 | -0.473 – 0.539 | .449 | 0.106 | 0.015 – 0.198 | **.012^*^** |
|  | **Go-RT** | | | | | | | | |
| Intercept | 413.925 | 398.398 – 429.452 | **<.001** | 412.191 | 396.829 – 427.552 | **<.001** | 410.648 | 394.748 – 426.548 | **<.001** |
| Years of smoking | 1.836 | 1.009 – 2.662 | **<.001^*^** | 1.885 | 1.063 – 2.706 | **<.001^*^** | 1.858 | 0.979 – 2.737 | **<.001^*^** |
| Sex (1: female) | 6.211 | -13.943 – 26.366 | .546 | 6.441 | -13.373 – 26.254 | .524 | 2.293 | -19.017 – 23.604 | .833 |
| IQ | -1.003 | -1.933 – -0.073 | **.034** | -0.931 | -1.858 – -0.004 | **.049** | -0.918 | -1.927 – 0.091 | .075 |
| Variable | 1.147 | -0.586 – 2.880 | .194 | 4.734 | -3.851 – 13.320 | .280 | -0.703 | -2.443 – 1.036 | .428 |
| Variable × Years of smoking | -0.118 | -0.203 – -0.034 | **.006** | -0.454 | -0.859 – -0.048 | **.028** | -0.039 | -0.133 – 0.056 | .423 |
| Variable × Sex | -0.460 | -2.627 – 1.706 | .677 | -0.665 | -11.506 – 10.175 | .904 | 0.874 | -1.309 – 3.057 | .433 |
| Variable × IQ | 0.034 | -0.077 – 0.145 | .545 | -0.280 | -0.800 – 0.239 | .290 | 0.032 | -0.077 – 0.140 | .565 |
| Stimulus type (1: smoking) | -0.953 | -2.819 – 0.913 | .317 | -0.961 | -2.828 – 0.905 | .313 | -1.095 | -2.968 – 0.778 | .252 |
| Variable × Stimulus type | 0.031 | -0.170 – 0.232 | .763 | 0.230 | -0.784 – 1.244 | .657 | 0.063 | -0.124 – 0.251 | .509 |

*Note*. The predictor *Variable* refers to either CPD, FTND, or QSU. For the effects of smoking-related variables(×stimulus type) on CE rates, one-sided *p*-values are reported, otherwise two-sided. All regression model predictors, except for sex, were grand-mean centered. CPD = Cigarettes Per Day; FTND = Fagerström Test for Nicotine Dependence; QSU = Questionnaire on Smoking Urges, brief version; CI = Confidence Interval; CE = Commission Error; Go-RT = mean Reaction Time in Go trials. ^*^ significant after Benjamini-Hochberg correction.

# Appendix E: Supplementary discussion

## Appendix E.1: OE rates

In terms of research questions 1 and 2, the smoking and control groups did not significantly differ in their OE rates. Regarding research question 3, the results for the moderating effect of age on the relationship between smoking-related variables and OE rates were inconclusive. In the smoking-specific GNGT, older individuals who smoked more CPD tended to exhibit somewhat lower OE rates, which is consistent with their tendentially faster RTs in Go trials. Meanwhile, younger, heavier smoking individuals tended to exhibit higher OE rates, which is consistent with their tendentially longer Go-RTs (speed-accuracy trade-off). However, in the general GNGT, the results are reversed and do not align with the observed performance pattern on CE rates and Go-RTs. More specifically, older individuals with higher severity of tobacco dependence and craving tended to show higher OE rates whereas younger individuals tended to show lower OE rates. Although speculative, the contradictory findings may be explained by the differing difficulty levels (overall higher CE rates were observed in the general GNGT) or contexts (smoking-related/neutral versus digits) of the two GNGTs. Future research should investigate group differences and the moderating effects of age by employing GNGTs with shorter response windows to achieve OE rates that are more sensitive to differentiating between participants.

## Appendix E.2: Years of smoking as predictor

The results of the regression models for research question 3 with smoking duration as predictor variable were found to be highly comparable to those of the models with age as predictor. As with age, a longer history of smoking was associated with lower CE rates. This is at odds with dual-process models of addiction, which posit that ongoing smoking promotes deficits in response inhibition. However, the design of the present study did not permit to distinguish the independent effects of the two predictors. Future research should examine individuals of the same age but varying smoking duration to test the assumption that a longer history of smoking leads to stronger deficits in response inhibition.

References

1. Heatherton TF, Kozlowski LT, Frecker RC, Fagerström KO. The Fagerström Test for Nicotine Dependence: a revision of the Fagerström Tolerance Questionnaire. *Br J Addict*. 1991;86(9):1119-1127. <http://doi.org/10.1111/j.1360-0443.1991.tb01879.x>.

2. Sheehan DV. *Mini International Neuropsychiatric Interview 7.0.2*. Medical Outcome Systems: Medical Outcome Systems; 2016.

3. Margraf J, Cwik JC. *Mini-DIPS Open Access: Diagnostisches Kurzinterview bei psychischen Störungen*; 2017.

4. Margraf J, Cwik JC, Pflug V, Schneider S. Strukturierte klinische Interviews zur Erfassung psychischer Störungen über die Lebensspanne. *Zeitschrift für Klinische Psychologie und Psychotherapie*. 2017;46(3):176-186. <http://doi.org/10.1026/1616-3443/a000430>.

5. Wright L, Lipszyc J, Dupuis A, Thayapararajah SW, Schachar R. Response inhibition and psychopathology: a meta-analysis of go/no-go task performance. *J Abnorm Psychol*. 2014;123(2):429-439. <http://doi.org/10.1037/a0036295>.

6. Luijten M, Littel M, Franken IHA. Deficits in inhibitory control in smokers during a go/nogo task: an investigation using event-related brain potentials. *PLoS One*. 2011;6(4):e18898. <http://doi.org/10.1371/journal.pone.0018898>.

7. Silva GM, Almeida NL, Souto JJS, Rodrigues SJ, Fernandes TP, Santos NA. Does chronic smoking affect performance on a go/no-go task? *Curr Psychol*. 2022;41(11):7636-7644. <http://doi.org/10.1007/s12144-020-01305-y>.

8. Tsegaye A, Guo C, Cserjési R, et al. Inhibitory performance in smokers relative to nonsmokers when exposed to neutral, smoking- and money-related pictures. *Behavioral sciences*. 2021;11(10):128. <http://doi.org/10.3390/bs11100128>.

9. Ling J, Heffernan T. The cognitive deficits associated with second-hand smoking. *Frontiers in Psychiatry*. 2016;7:46. <http://doi.org/10.3389/fpsyt.2016.00046>.

10. Faul F, Erdfelder E, Lang A-G, Buchner A. G*Power 3: a flexible statistical power analysis program for the social, behavioral, and biomedical sciences. *Behav Res Methods*. 2007;39(2):175-191. <http://doi.org/10.3758/bf03193146>.

11. Smith JL, Mattick RP, Jamadar SD, Iredale JM. Deficits in behavioural inhibition in substance abuse and addiction: a meta-analysis. *Drug Alcohol Depend*. 2014;145:1-33. <http://doi.org/10.1016/j.drugalcdep.2014.08.009>.

12. Motka F, Wittekind CE, Ascone L, Kühn S. Efficacy and working mechanisms of a Go/No-Go task-based inhibition training in smoking: A randomized-controlled trial. *Behav Res Ther*. 2025;185(104672). <http://doi.org/10.1016/j.brat.2024.104672>.

13. Hedge C, Powell G, Sumner P. The reliability paradox: Why robust cognitive tasks do not produce reliable individual differences. *Behav Res Methods*. 2018;50(3):1166-1186. <http://doi.org/10.3758/s13428-017-0935-1>.

14. Parsons S, Kruijt A-W, Fox E. Psychological science needs a standard practice of reporting the reliability of cognitive-behavioral measurements. *Advances in Methods and Practices in Psychological Science*. 2019;2(4):378-395. <http://doi.org/10.1177/2515245919879695>.

15. Logan GD, Cowan WB. On the ability to inhibit thought and action: A theory of an act of control. *Psychological Review*. 1984;91(3):295-327. doi:10.1037/0033-295X.91.3.295.

16. Wiers CE, Kühn S, Javadi AH, et al. Automatic approach bias towards smoking cues is present in smokers but not in ex-smokers. *Psychopharmacology*. 2013;229(1):187-197. <http://doi.org/10.1007/s00213-013-3098-5>.

17. Luijten M, Veltman DJ, van den Brink W, et al. Neurobiological substrate of smoking-related attentional bias. *Neuroimage*. 2011;54(3):2374-2381. <http://doi.org/10.1016/j.neuroimage.2010.09.064>.

18. Wittekind CE, Takano K, Sckopke P, et al. Efficacy of approach bias modification as an add-on to smoking cessation treatment: study protocol for a randomized-controlled double-blind trial. *Trials*. 2022;23(1):223. <http://doi.org/10.1186/s13063-022-06155-6>.

19. Jakobsen JC, Gluud C, Wetterslev J, Winkel P. When and how should multiple imputation be used for handling missing data in randomised clinical trials – a practical guide with flowcharts. *BMC Med Res Methodol*. 2017;17(1):1-10. <http://doi.org/10.1186/s12874-017-0442-1>.

20. *splithalfr: Estimates split-half reliabilities for scoring algorithms of cognitive tasks and questionnaires:* Zenodo; 2023.

21. Detandt S, Bazan A, Schröder E, et al. A smoking-related background helps moderate smokers to focus: An event-related potential study using a Go-NoGo task. *Clinical neurophysiology*. 2017;128(10):1872-1885. <http://doi.org/10.1016/j.clinph.2017.07.416>.

22. Li X, Li W, Chen H, Cao N, Zhao B. Cigarette-specific disgust aroused by smoking warning images strengthens smokers’ inhibitory control under smoking-related background in go/nogo task. *Psychopharmacology (Berl)*. 2021;238(10):2827-2838. <http://doi.org/10.1007/s00213-021-05898-5>.

23. Liu C, Dong F, Li Y, et al. 12 h abstinence-induced ERP changes in young smokers: Electrophysiological evidence from a Go/NoGo study. *Front Psychol*. 2019;10:1814. <http://doi.org/10.3389/fpsyg.2019.01814>.

24. Yu Z, Guindani M, Grieco SF, Chen L, Holmes TC, Xu X. Beyond t test and ANOVA: applications of mixed-effects models for more rigorous statistical analysis in neuroscience research. *Neuron*. 2022;110(1):21-35. <http://doi.org/10.1016/j.neuron.2021.10.030>.

25. Field AP, Wilcox RR. Robust statistical methods: A primer for clinical psychology and experimental psychopathology researchers. *Behav Res Ther*. 2017;98:19-38. <http://doi.org/10.1016/j.brat.2017.05.013>.

26. Mair P, Wilcox R. Robust statistical methods in R using the WRS2 package. *Behav Res Methods*. 2020;52(2):464-488. <http://doi.org/10.3758/s13428-019-01246-w>.

27. Wilcox RR, Rousselet GA. An updated guide to robust statistical methods in neuroscience. *Current Protocols*. 2023;3(3):e719. <http://doi.org/10.1002/cpz1.719>.

28. *BayesFactor version 0.9.9: An R package for computing Bayes factor for a variety of psychological research designs*; 2014.

29. Lee MD, Wagenmakers E-J. *Bayesian cognitive modeling: A practical course*. Cambridge: Cambridge University Press; 2013.

30. Venables WN, Ripley BD. *Modern applied statistics with S.* 4^th^ ed. New York: Springer; 2002. Statistics and computing.

31. Koller M. robustlmm: An R package for robust estimation of linear mixed-effects models by Manuel Koller. *Journal of Statistical Software*. 2016;75(6):1-24.

32. Benjamini Y, Hochberg Y. Controlling the False Discovery Rate: A practical and powerful approach to multiple testing. *Journal of the Royal Statistical Society Series B: Statistical Methodology*. 1995;57(1):289-300. <http://doi.org/10.1111/j.2517-6161.1995.tb02031.x>.

33. Blakesley RE, Mazumdar S, Dew MA, et al. Comparisons of methods for multiple hypothesis testing in neuropsychological research. *Neuropsychology*. 2009;23(2):255-264. <http://doi.org/10.1037/a0012850>.

34. Vickerstaff V, Omar RZ, Ambler G. Methods to adjust for multiple comparisons in the analysis and sample size calculation of randomised controlled trials with multiple primary outcomes. *BMC Med Res Methodol*. 2019;19(1):129. <http://doi.org/10.1186/s12874-019-0754-4>.

35. Long JA. *Comprehensive, user-friendly toolkit for probing interactions [R package interactions version 1.1.5]:* Comprehensive R Archive Network (CRAN); 2021.

36. Bates D, Mächler M, Bolker B, Walker S. Fitting linear mixed-effects models using lme4. *J. Stat. Soft.* 2015;67(1). <http://doi.org/10.18637/jss.v067.i01>.

1. The recruitment of participants commenced in May 2018. Due to the corona pandemic and the closing of university buildings, recruitment was suspended in November 2020, with *n* = 79 participants having been enrolled up to that point. Recruitment resumed in January 2023, and the final sample size of *N* = 122 was reached in October 2023. [↑](#footnote-ref-1)
2. Thereby, we followed the pre-processing procedure as used for other reaction time tasks within the field of addiction research, see Wittekind et al. [18]. [↑](#footnote-ref-2)
3. Robust explanatory effect size for *t*-tests on means (small effect: *ξ* = .10, medium effect: *ξ* = .30, see [26]. [↑](#footnote-ref-3)
4. 3 < BF_10_ < 10: indicates substantial evidence in favour of a difference between groups; 1 < BF_10_ < 3: indicates weak evidence in favour of a difference between groups; 1/3 < BF_10_ < 1: indicates weak evidence in favour of no difference between groups; 1/10 < BF_10_ < 1/3: indicates substantial evidence in favour of no difference between groups, see [29]. [↑](#footnote-ref-4)
